# Salivary Cortisol and Cognitive Decline and Alzheimer Disease in Older Adults

**DOI:** 10.1001/jamanetworkopen.2026.22955

**Published:** 2026-07-15

**Authors:** Ted K. S. Ng, Todd Beck, Yashwanth Sudhini, Robert S. Wilson, Denis A. Evans, Kumar B. Rajan

**Affiliations:** 1 Rush Institute for Healthy Aging, Rush University Medical Center, Chicago, Illinois; 2 Rush Alzheimer’s Disease Center, Rush University Medical Center, Chicago, Illinois

## Abstract

**Importance:**

Alterations in hypothalamic-pituitary-adrenal axis activity have been implicated in cognitive aging, yet large, racially diverse studies using physiologically active salivary cortisol studies are limited.

**Objective:**

To determine whether diurnal salivary cortisol indices are associated with cognitive outcomes, including incident Alzheimer disease (AD), and whether distributions and associations differ by race.

**Design, Setting, and Participants:**

This prospective cohort study was conducted from 2000 to 2011, with 11 years of follow-up, as part of the Chicago Health and Aging Project, a community-based study of older adults in urban Chicago. Participants provided salivary cortisol samples across a day (waking, afternoon, and bedtime) and completed cognitive assessments. Analyses were conducted from June to October 2025.

**Exposures:**

Five salivary cortisol indices capturing intraday variability (coefficient of variation [CV]), cumulative exposure (mean cortisol and area under the curve with respect to ground [AUCg]), and diurnal change (diurnal slope and AUC with respect to increase [AUCi]).

**Main Outcomes and Measures:**

Outcomes of interest were global cognition, cognitive decline, and incident AD, adjusted for demographic, health, and behavioral factors.

**Results:**

Among 3895 participants (mean [SD] age, 76.7 [6.8] years; 2509 [64.4%] women; 2503 Black participants [64.3%] and 1392 White participants [35.7%]), all cortisol indices were cross-sectionally associated with cognitive performance. Longitudinally, compared with the first quintile (Q1), medium to high cortisol intraday variability (CV) was associated with slower decline (Q3: β = 0.02 [95% CI, 0.001 to 0.04]; *P* = .04; Q4: β = 0.03 [95% CI, 0.01 to 0.04]; *P* = .003), whereas the highest cumulative exposure was associated with faster decline (mean cortisol Q5: β = −0.02 [95% CI, −0.04 to −0.004]; *P* = .02; AUCg Q5: β = −0.02 [95% CI, −0.03 to 0.00]; *P* = .046). Diurnal change indices (slope, AUCi) were not associated with decline or incident AD. Black participants exhibited lower cortisol exposure but flatter slopes and lower intraday variability, reflecting a blunted diurnal rhythm; associations with decline were similar across races.

**Conclusions and Relevance:**

In this cohort study of Black and White older adults, altered diurnal cortisol patterning was associated with faster cognitive decline but not short-term AD risk. These findings support salivary cortisol as a potential early biomarker of neurocognitive aging. Racial differences in cortisol patterning suggest differential biological embedding of stress, consistent with structural inequities.

## Key Points

**Question:**

Are diurnal salivary cortisol indices associated with cognitive performance, decline, and Alzheimer disease (AD), and do these associations differ by race?

**Findings:**

In a cohort study of 3895 Black and White older adults, indices reflecting altered diurnal cortisol patterning and cumulative exposure were associated with cognitive decline. Black participants exhibited lower cumulative cortisol levels, lower intraday variability, and flatter diurnal slopes, reflecting a blunted diurnal rhythm, yet associations between cortisol and cognitive outcomes were similar across racial groups.

**Meaning:**

These findings suggest that salivary cortisol may capture early, modifiable stress physiology associated with cognitive aging and racial differences in diurnal cortisol patterning.

## Teaser

This cohort study examines whether diurnal salivary cortisol indices are associated with cognitive outcomes, including incident Alzheimer disease, and whether distributions and associations differ by race.

## Introduction

Alzheimer disease (AD) is a leading global health challenge, contributing substantially to disability, dependency, and mortality in older adults.^1,2,3^ As populations age, the burden of AD is rapidly escalating, underscoring the need to expand biomarker discovery beyond the established amyloid, tau, and neurodegeneration (AT[N]) framework to encompass an X dimension that reflects systemic processes, such as inflammation, metabolism, and stress physiology,^4^ that may shape cognitive aging. Within this context, stress can be conceptualized as a multifaceted and modifiable process operating across the life course, arising from structured exposures across individual, interpersonal, and broader social contexts, and influencing health through behavioral pathways and cumulative biological embedding. Frameworks such as allostatic load^5,6^ and dementia prevention models, including the 2024 Lancet Commission,^7^ highlight how stress-related processes overlap with and may contribute to established modifiable risk factors for cognitive decline. In this context, salivary cortisol provides a physiologically grounded, population-feasible marker of hypothalamic-pituitary-adrenal (HPA) axis activity, capturing one biological pathway through which stress-related processes may influence neurocognitive aging.

The HPA axis regulates cortisol secretion, which modulates stress responsivity and maintains homeostasis.^8,9,10^ Cortisol follows a circadian rhythm marked by a sharp postawakening rise and a gradual decline across the day.^11^ Because cortisol readily crosses the blood-brain barrier and binds receptors in regions critical for cognition, including the hippocampus and prefrontal cortex,^12,13^ alterations in cortisol levels may contribute to neurodegeneration and cognitive decline.

Elevated peripheral cortisol has been associated with hippocampal atrophy, impaired cerebral metabolism, and increased AD risk.^14,15,16^ However, findings on peripheral cortisol and cognitive outcomes have been conflicting. Meta-analyses indicate that higher blood cortisol is associated with progression in mild cognitive impairment and early AD,^13^ yet associations in cognitively healthy older adults are mixed, with some reports paradoxically reporting higher blood cortisol is associated with better cognition.^17^ Furthermore, existing evidence is limited by cross-sectional designs, small or homogeneous clinical samples, and a lack of comprehensive diurnal profiling that captures complementary yet distinct dimensions of HPA axis physiology. Nonlinear associations are also rarely examined, and few studies track longitudinal cognitive change or incident AD. Although prior studies have examined salivary cortisol in association with cognitive decline, these have typically focused on cognitive outcomes alone and have rarely evaluated incident AD within large, population-based cohorts. Moreover, racial minority groups, despite bearing a disproportionate burden of AD-related dementia,^18,19^ remain underrepresented. Specifically, Black adults are disproportionately exposed to psychosocial and structural stressors, partly due to structural inequities,^20,21,22^ yet data on racial differences in diurnal cortisol patterns and their cognitive implications remain sparse.

Notably, distinct from available biofluids, such as blood and cerebrospinal fluid (CSF), salivary cortisol offers distinct advantages for large population studies: it is noninvasive, low-cost, and stress-free and minimally burdensome to collect. Importantly, unlike CSF or blood cortisol, which contain both bound (inactive) and unbound (active) fractions, saliva reflects physiologically active free cortisol, making it a specific marker of biologically active cortisol exposure and thus particularly valuable for research on stress and cognitive aging.^13^ Furthermore, the feasibility of repeated intraday salivary sampling enables assessment of diurnal variation, capturing dynamic aspects of HPA axis function that are not reflected in a single blood or CSF measure typically used in AD research. However, even AD studies that have examined salivary cortisol rarely derived diurnal indices commonly used in psychoneuroendocrinological research, such as area under the curve (AUC) measures. These indices, ie, AUC with respect to ground (AUCg) and AUC with respect to increase (AUCi), quantify day-long total cortisol output and diurnal change, respectively, providing a more accurate and comprehensive characterization of HPA-axis activity than single time point, mean, or ratio-based cortisol metrics.^23,24,25,26^

To address these gaps, we comprehensively characterized diurnal salivary cortisol in a large, racially diverse, community-based cohort of nearly 4000 older adults. We derived 5 indices of diurnal HPA axis activities and examined their associations with baseline cognition, and prospectively with cognitive decline and incident AD. We also evaluated these associations across Black and White participants to assess potential differences in biological embedding and cortisol-cognition associations.

## Methods

This cohort study was conducted as part of the Chicago Health and Aging Project (CHAP), approved by the institutional review board of Rush University Medical Center, and all participants gave written informed consent. This study adheres to the Strengthening the Reporting of Observational Studies in Epidemiology (STROBE) reporting guideline for cohort studies.

### Study Participants

Data were drawn from the CHAP, an established population-based longitudinal study of aging and AD among community-dwelling older adults on the South Side of Chicago in 4 Chicago neighborhoods with substantial proportions of Black and White older adults.^27,28^ CHAP data were collected in triennial cycles, with the baseline cohort starting in 1993 and subsequent cycles in 1997, 2000, 2003, 2006, 2009, and 2012. In each cycle, a stratified random sample based on age, sex, race, and cognitive level was selected for clinical evaluation of incident AD when the participants also provided blood samples. Saliva sampling was initiated in CHAP cycle 5 (2006), during which most cortisol measures were collected. A smaller subset of cortisol measures was obtained from earlier saliva collections conducted in a nested caregiver substudy among CHAP participants. Caregiver-only participants were not included in the present analysis.

### Salivary Sample Collection and Handling

Participants were given instructions to self-collect saliva samples. Samples were collected in color-coded sterile cotton swabs and tubes: green (morning), immediately on awakening and before getting out of bed, with the exact wake time and the time sample was obtained recorded; pink (afternoon), between 5 and 6 pm, at least 1 hour before having dinner; and blue (evening), between 9 and 10 pm or immediately before bedtime if earlier than 9 pm. Participants recorded the exact collection time on a provided card and refrigerated tubes until mailing or pickup. They were instructed to avoid food, drink, tooth brushing, or mouthwash for at least 1 hour prior to sampling and were encouraged to collect all 3 samples on the same day, with missed samples collected the following day. For all time points, participants recorded the collection time on a card, stored the samples in a refrigerator after each collection, and returned all materials in a preaddressed, stamped envelope for mailing or pickup. Participants were provided with contact information for study staff to address any questions and received a follow-up call the next day to ensure protocol adherence. On receipt in the laboratory, samples were logged, centrifuged, aliquoted if necessary, and stored at −80 °C until assay. Recorded collection times were used for quality control to assess protocol adherence and identify samples with clear nonadherence, and were not incorporated into analytic models. Cortisol indices were calculated using available samples without imputation; participants with at least 1 valid sample were included. Although multiday sampling may improve reliability of certain cortisol indices,^29^ particularly diurnal slope, such protocols are often not feasible in large population-based studies, and single-day multi–time point sampling remains widely used in large-scale epidemiologic research.^17,24,30,31,32^

### Salivary Cortisol Measurement and Calculation

**Figure 1.**
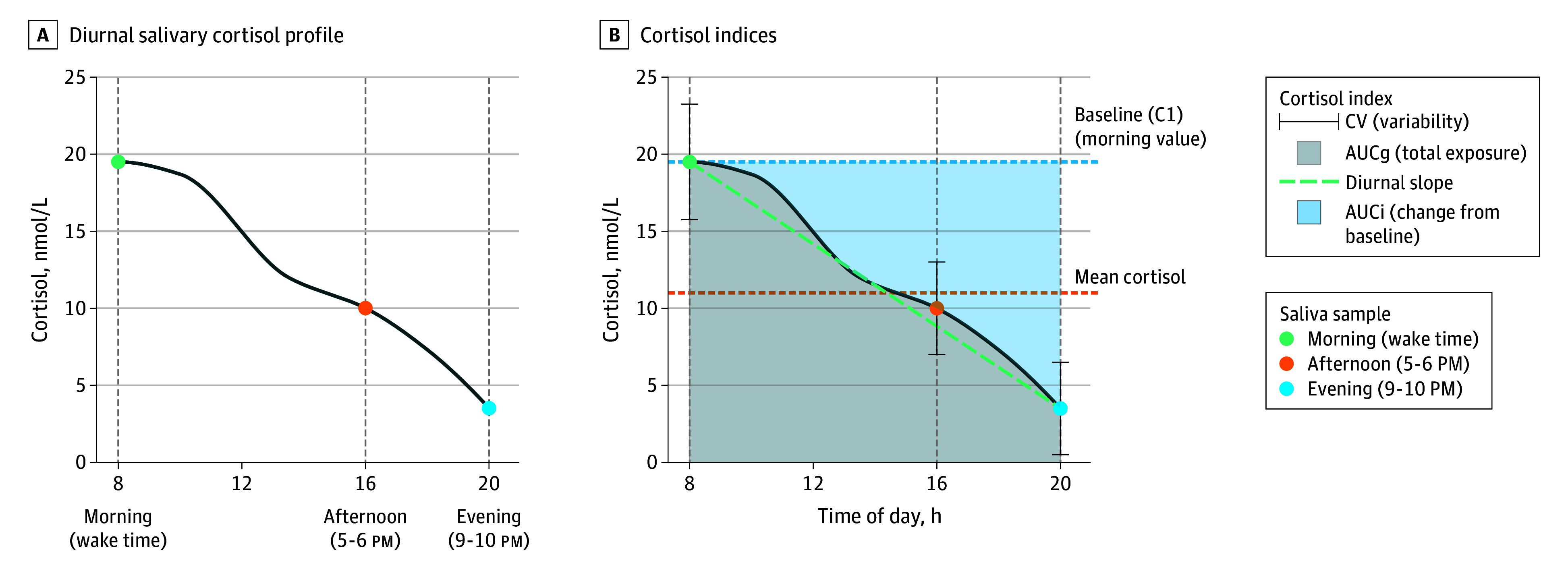
Schematic Representation of Diurnal Salivary Cortisol Profiles and 5 Derived Indices A, Representative diurnal cortisol rhythm derived from morning, afternoon, and evening saliva samples. B, Illustration of the 5 analytic indices: coefficient of variation (CV; intraday variability), area under the curve with respect to ground (AUCg; total daily cortisol output), AUC with respect to increase (AUCi; diurnal change relative to baseline), mean cortisol (basal level), and diurnal slope (rate of decline). AUCi is depicted as the signed area between the diurnal cortisol curve and the morning baseline, with hatched regions above and below the baseline denoting positive and negative contributions, respectively. CV reflects the cumulative deviation of each time point from the daily mean, indexing dispersion across sampled diurnal cortisol value. Definitions, physiological interpretations, and analytic roles of each index are summarized in eTable 1 in Supplement 1.

Baseline salivary cortisol was assayed using enzyme-linked immunosorbent assay (Salimetrics) according to standardized protocols at a certified laboratory. The mean of duplicate measurements was used for each participant (coefficient of variation [CV], 7.3%). Extreme outlying values (cortisol >700 nmol/L) or samples indicating protocol nonadherence were excluded. Based on cortisol levels from the 3 collection time points, 5 complementary indices of diurnal HPA axis activity were derived to capture intraday variability, total daily output, and diurnal change (Figure 1A and B). Following prior literature and biological rationale, intraday variability (CV) was designated the primary index, cumulative exposure (mean cortisol and AUCg) as secondary indices, and diurnal change (slope and AUCi) as exploratory indices. This framework aligns analytic priorities with underlying physiological dimensions (eMethods and eTable 1 in Supplement 1). Salivary cortisol data were cleaned prior to analysis, including correction of sampling times when clearly identifiable and exclusion of implausible values (cortisol >700 nmol/L).

### Cognitive Assessment

Longitudinal cognitive function was assessed during in-home interviews at 3-year intervals using 4 standardized tests: immediate and delayed recall of the East Boston Story (episodic memory),^33^ the oral Symbol Digit Modalities Test (perceptual speed and executive function),^34^ and the Mini-Mental State Examination (global cognition).^35^ These instruments were chosen for their strong psychometric properties, sensitivity to AD-related dementia–related domains, and feasibility in large, community-based cohorts, including in CHAP.^36^ This protocol follows established methods from CHAP, in which these measures demonstrated excellent validity and reliability.^27,36,37^ A prior CHAP factor analysis^36^ indicated that all tests loaded on a single factor explained 75% of variance. Therefore, a composite global cognition score was derived by *Z*-standardizing each test to the baseline mean and SD and calculating the mean across tests, with higher scores indicating better cognition.^36^ Composite scores are preferred in longitudinal studies to minimize measurement error and ceiling or floor effects.^36^ Cognitive decline was modeled using repeated measures across study waves.

### Clinical AD Outcomes

Incidence of AD was ascertained via clinical evaluations in a stratified random sample each study cycle, following standardized neurological and cognitive assessments, with diagnoses made by a neurologist blinded to test scores using National Institute of Neurological and Communicative Disorders and Stroke and the Alzheimer Disease and Related Disorders Association criteria.^27,37,38^

### Covariates

Models were adjusted for demographics, including baseline age, education (years of schooling) (centered at 75 years and 12 years, respectively), sex, and self-reported race (Black and White), assessed using data format from the 1990 US Census Bureau.^39^ Additional covariates were *APOE ε4* status; self-reported chronic conditions, including heart disease, diabetes, hypertension, and stroke, identified using self-report items from the Established Populations for the Epidemiologic Study of the Elderly^40^; medications, which were physically observed and recorded by the interviewers (ie, antidepressant and steroid use, including glucocorticoids, inhaled steroids, and oral steroids); body mass index (BMI); smoking status; and alcohol consumption. Effect modification by race (Black or White) was assessed through interaction and stratified analyses.

### Statistical Analysis

Baseline descriptive statistics were computed for demographic and cognitive characteristics, stratified by race (Black or White). Medians and IQRs were reported. Descriptive comparisons between Black and White participants were based on *t* tests for untransformed continuous variables, χ^2^ tests for categorical variables, and Wilcoxon rank tests for cortisol indices.

Given the inverse *U*-shaped associations and threshold effects hypothesized from physiological roles (eTable 1 in Supplement 1), primary analyses modeled baseline cortisol indices in quintiles (with the lowest quintile [Q1] as the reference group) to examine associations with baseline cognitive performance, rate of cognitive decline, and incident AD risk. Cortisol indices were constructed using available data with prespecified requirements. Mean cortisol required at least 1 valid sample (100% available), coefficient of variation (CV) required at least 2 samples (95%), and AUC measures (AUCg and AUCi) and diurnal slope required at least 2 samples with valid time-of-day information (93%). Indices were calculated without imputation using standard formulas appropriate to the number and timing of available samples. Participants with insufficient data for a given index were excluded from analyses of that outcome, resulting in minimal variation in analytic sample size (0%-7% exclusion depending on the index), as reflected in the samples sizes reported in eTable 3 and eTable 4 in Supplement 1.

Mixed-effects models with random intercepts and slopes were used to estimate cognitive trajectories over time. Time since baseline cortisol assessment, in years, was used to capture annual rates of cognitive change. Discrete-time survival analysis, implemented via pooled logistic regression, evaluated AD incidence within the adjudicated subsample, reflecting the interval-censored structure of diagnoses assessed at discrete follow-up waves. Given the long preclinical phase of AD and the reduced sample size, incident AD analyses were considered exploratory.

Supplementary analyses treated baseline cortisol indices as linear continuous independent variables in models, with quadratic terms further added to test for U-shaped associations. Hierarchical regression models sequentially incorporated covariates. Model 1 controlled for demographics (age, sex, education, and race). Model 2 additionally included health factors (chronic conditions, BMI, *APOE4* status, and medications, including antidepressants and glucocorticoids). Model 3 further added health behaviors (smoking and alcohol).

To evaluate race as a potential modifier, we conducted 2 complementary analyses. First, 3-way interaction models including race × time × cortisol index terms (modeled in quintiles) were fitted to test differential longitudinal associations. Second, we stratified the sample by race (Black and White).

All regression models were implemented in SAS software version 9.4 (SAS Institute), and graphical representations were generated with R software version 4.5 (R Project for statistical Computing). Two-sided *P* < .05 was considered statistically significant. As all analyses were based on a priori hypotheses with prespecified primary, secondary, and exploratory indices and contrasts based on a single regression model for each cortisol index, no adjustments for multiple comparisons were applied.

Sensitivity analysis included analysis using mixed-effects models to test the robustness of conclusions excluding baseline cases in the lowest tenth percentile of global cognitive performance, consistent with our prior analyses on CHAP.^41,42,43^ All analyses were conducted from June to October 2025.

## Results

### Sample Characteristics

Of 5008 eligible individuals, 4618 accepted saliva collection kits and 3898 returned at least 1 valid cortisol sample. After excluding 3 individuals missing education data, 3895 participants (mean [SD] age, 76.7 [6.8] years; 2509 [64.4%] women; 2503 Black participants [64.3%] and 1392 White participants [35.7%]) were included in the primary baseline cortisol-longitudinal cognitive outcome models (eFigure 1 in the Supplement 1). A subset of 825 participants had clinical evaluations available for incident AD analyses. Among participants with valid cortisol data, 3243 (83%) provided 3 samples, 468 (12%) provided 2 samples, and 184 (5%) provided 1 sample. Baseline demographic and clinical characteristics by race are shown in the Table.

**Table.** Baseline Participant Demographics

Characteristic	Overall (n = 3895)	Black participants (n = 2503)	White participants (n = 1392)	*P* value^a^
Age, mean (SD), y	76.7 (6.8)	75.8 (6.3)	78.4 (7.4)	<.001
Sex, No. (%)				
Female	2509 (64)	1633 (65)	876 (63)	.16
Male	1386 (36)	870 (35)	516 (37)	
Education, mean (SD), y	13.0 (3.3)	12.1 (3.1)	14.4 (3.1)	<.001
Cortisol CV, mean (SD)	71.3 (35.2)	66.1 (34.9)	80.7 (33.9)	<.001
Cortisol, median (IQR), nmol/L	7.3 (5.1 to 10.7)	7.1 (4.9 to 10.5)	7.7 (5.3 to 11.0)	.002
AUCg, median (IQR)	7.7 (5.3 to 11.0)	7.3 (5.1 to 10.7)	8.3 (5.7 to 11.5)	<.001
Diurnal slope, mean (SD)	−0.074 (0.028)	−0.071 (0.027)	−0.080 (0.029)	<.001
AUCi, median (IQR)	−4.7 (−9.7 to −1.0)	−3.8 (−8.2 to −0.6)	−6.9 (−12.2 to −2.2)	<.001
Baseline global cognition, mean (SD)	0.38 (0.69)	0.24 (0.70)	0.63 (0.60)	<.001
Rate of cognitive decline^3^, mean (SD)	−0.05 (0.28)	−0.06 (0.19)	−0.04 (0.21)	.02
BMI, mean (SD)	28.2 (6.1)	28.9 (6.3)	27.0 (5.4)	<.001
Medical conditions, mean (SD), No.	1.47 (1.05)	1.56 (1.05)	1.32 (1.03)	<.001
Antidepressant use, No. (%)	247 (6.3)	96 (3.8)	151 (11)	<.001
Steroid use, No. (%)	216 (5.5)	127 (5.1)	89 (6.4)	.10
Alcohol use, No. (%)				
Moderate	1033 (27)	401 (16)	632 (45)	<.001
High	216 (5.5)	72 (2.9)	144 (10)	<.001
*APOE4* carrier, No. (%)	1174 (33)	848 (38)	326 (25)	<.001
Follow-up time, mean (SD), y	2.61 (2.22)	2.63 (2.35)	2.56 (1.99)	.33

Abbreviations: AUCg, area under the curve, ground; AUCi, area under the curve, increase; CV, coefficient of variation; BMI, body mass index (calculated as weight in kilograms divided by height in meters squared).

^a^ Welch 2-sample *t* test; Pearson χ^2^ test; Wilcoxon rank sum test.

**Figure 2.**
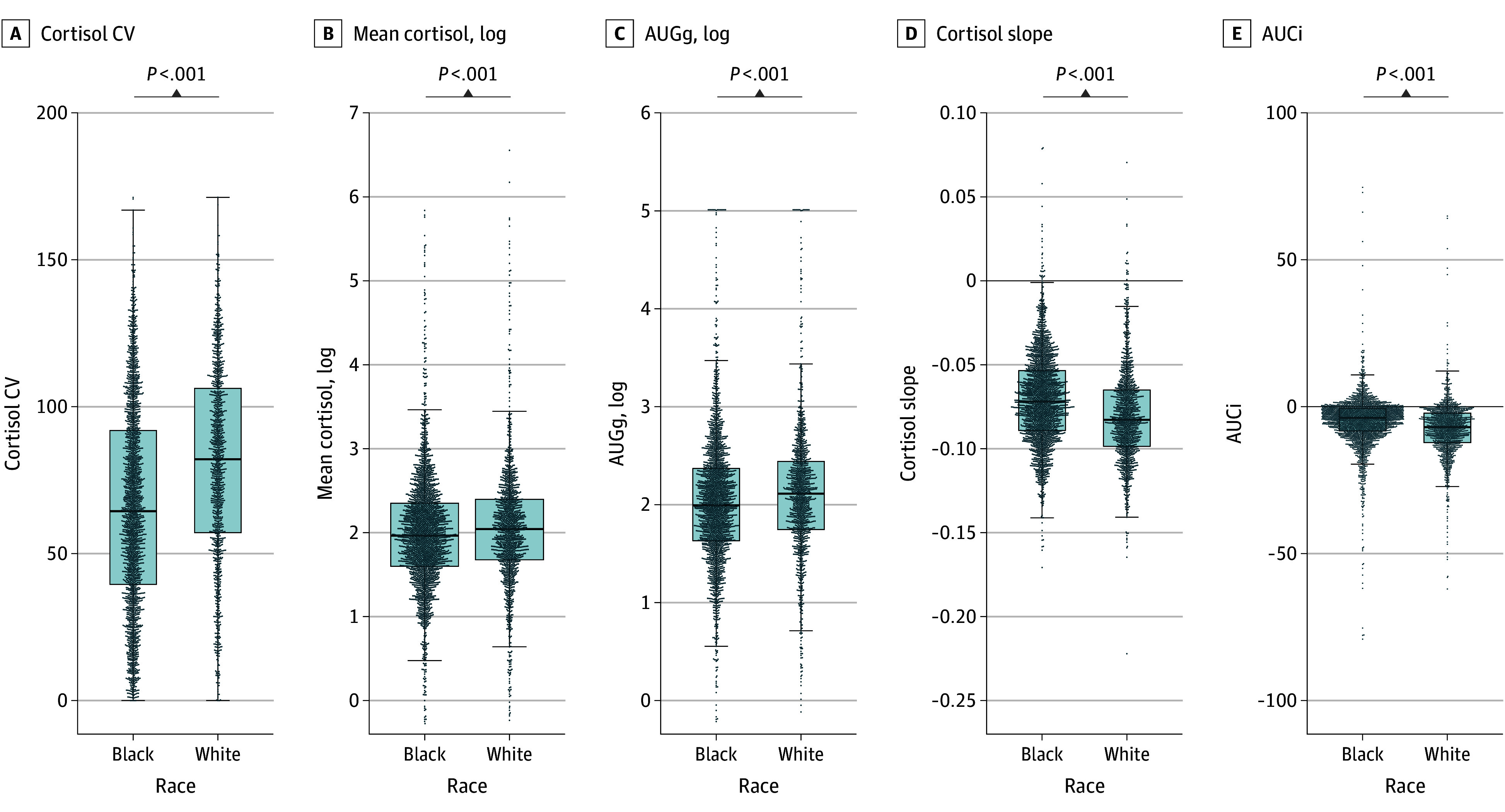
Bee-Swarm With Box-and-Whisker Plots of Distribution of Person-Specific Diurnal Salivary Cortisol Indices by Race Across all 5 cortisol indices, White participants exhibited higher cortisol variability (CV) and greater cumulative cortisol output (mean cortisol and area under the curve with respect to ground [AUCg]), whereas Black participants showed lower CV, flatter diurnal slopes, and lower AUC with respect to increase (AUCi), reflecting attenuated intraday fluctuation in hypothalamic-pituitary-adrenal axis activity. Bold lines indicate medians; boxes, IQRs; whiskers, median ± 1.5 × IQR.

Baseline comparisons indicated that, compared with White participants, Black participants exhibited lower cortisol variability (mean [SD] CV, 66.1 [34.9] vs 80.7 [33.9]; *P* < .001), lower cumulative cortisol output (median [IQR] cortisol, 7.1 [4.9-10.5] nmol/L vs 7.7 [5.3-11.0] nmol/L; *P* = .002; median [IQR] AUCg, 7.3 [5.1-10.7] vs 8.3 [5.7-11.5]; *P* < .001), and attenuated diurnal changes (mean [SD] slope, –0.071 [0.027] vs –0.080 [0.029]; *P* < .001; median [IQR] AUCi, –3.8 [–8.2 to –0.6] vs –6.9 [–12.2 to –2.2]; *P* < .001) (Figure 2 and Table). Regarding cognitive outcomes, baseline global cognition was lower in Black participants (mean [SD] score, 0.24 [0.70] vs 0.63 [0.60]; *P* < .001), and the rate of cognitive decline was also faster among Black compared with White adults (β = –0.06 [95% CI, –0.43 to 0.31] vs β = –0.04 [95% CI, –0.45 to 0.37]; *P* = .02).

eTable 2 in Supplement 1 focuses on the AD subsample of 825 participants, including 92 participants (11.2%) who developed incident AD. Participants who developed incident AD showed lower cortisol intraday variability and a blunted diurnal cortisol rhythm, reflected by lower CV, higher AUCi, and flatter diurnal slope.

### Cross-Sectional and Longitudinal Associations of Salivary Cortisol Quintiles With Cognition

**Figure 3.**
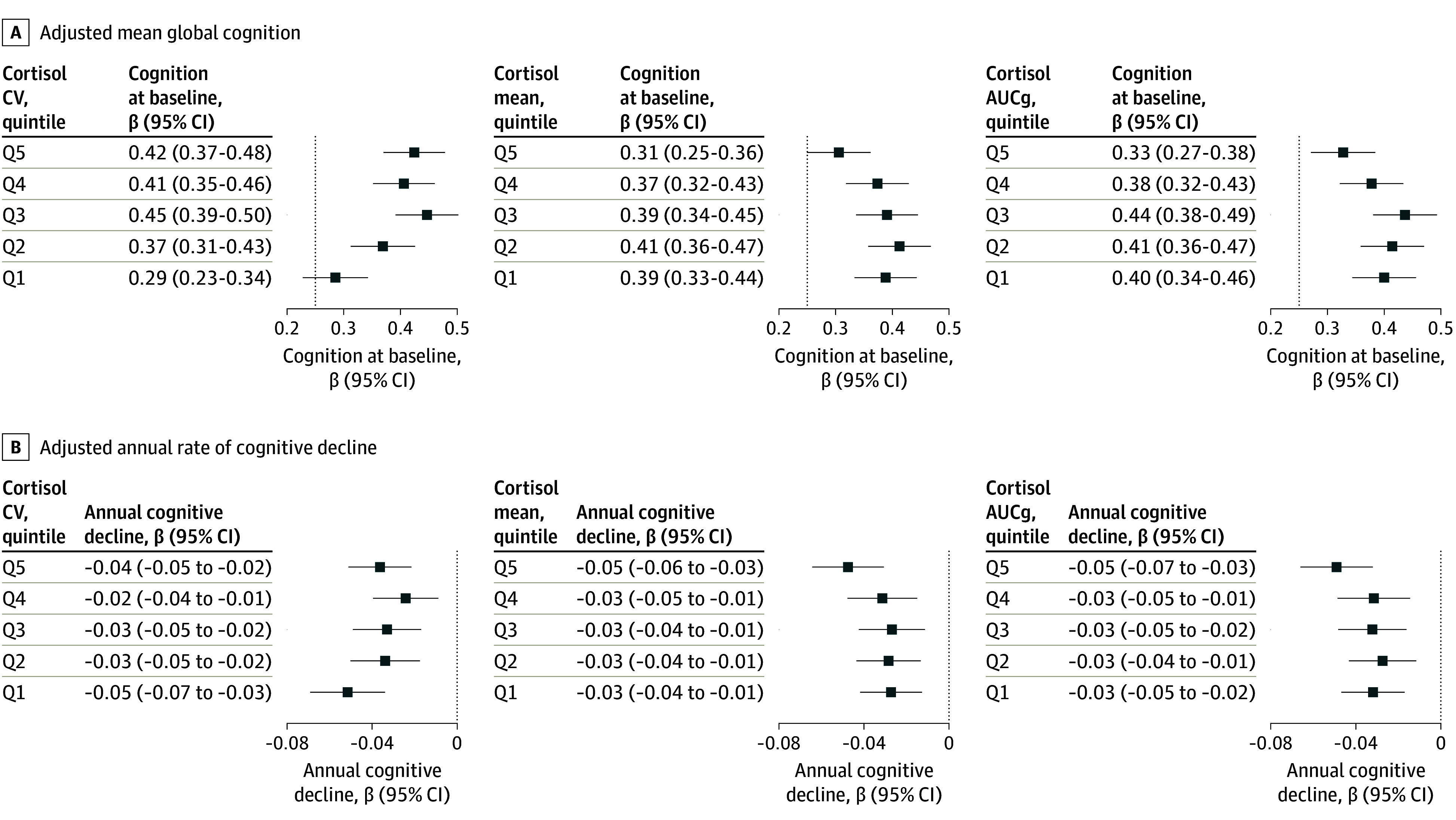
Dot Plots of Adjusted Mean Global Cognition at Baseline and Annual Rate of Cognitive Decline β values are given as standardized units. A, All indices demonstrated curvilinear associations. Higher intraday cortisol variability (CV; Q2-Q5) was significantly associated with better cognitive performance at baseline. Higher cumulative exposures (mean, area under the curve with respect to ground [AUCg]) were associated with poorer cognition at the highest quintile (Q5). B, Curvilinear associations and significance patterns mirrored those observed at baseline. Moderate intraday CV was associated with slower cognitive decline, whereas higher cumulative exposure (mean cortisol, AUCg) was associated with faster decline. Models adjusted for age, sex, race, education, body mass index, comorbidities, medication use, *APOE ε4* status, smoking, and alcohol use.

Full quintile results are presented in eTable 3 and eFigure 2 in Supplement 1 and Figure 3A-B. All cross-sectional and longitudinal results presented here are based on model 3, the fully adjusted model.

#### Intraday Variability (CV)

In fully adjusted models, compared with Q1, middle to high CV (Q3-Q4) was associated with higher baseline cognition (Q3: β = 0.16 [95% CI, 0.10-0.22], *P* < .001; Q4: β = 0.12 [95% CI, 0.06-0.18]; *P* < .001) and slower decline (Q3 × time: β = 0.02 [95% CI, 0.0004-0.04]; *P* = .04; Q4 × time: β = 0.03 [95% CI, 0.009-0.05], *P* = .003). The highest quintile (Q5) also was associated with higher baseline cognition (β = 0.14 [95% CI, 0.08-0.20]; *P* < .001) but not with decline.

#### Mean Cortisol and Total Daily Output (AUCg)

Compared with Q1, Q5 of mean cortisol was associated with lower baseline cognition (β = –0.08 [95% CI, –0.14 to –0.02]; *P* = .005) and faster decline (β = –0.02 [95% CI, –0.04 to –0.004]; *P* = .02). Compared with Q1, the highest quintile (Q5) of AUCg was associated with lower baseline cognition (β = –0.07 [95% CI, –0.13 to –0.01]; *P* = .02) and faster decline (β = –0.02 [95% CI, –0.03 to 0.00]; *P* = .046).

#### Diurnal Slope and Change Relative to Baseline (AUCi)

Compared with Q1, Q4 and Q5 diurnal slopes were associated with lower baseline cognition (Q5: β = –0.14 [95% CI, –0.20 to –0.08]; *P* < .001) but not with longitudinal decline. Compared with Q1, Q5 AUCi was associated with lower baseline cognition (β = –0.12 [95% CI, –0.18 to –0.06]; *P* < .001) but was not associated with longitudinal decline.

#### Supplementary Continuous and Quadratic Models

Continuous models yielded convergent patterns with the quintile analyses. For example, higher AUCg was associated with lower baseline cognition (β = –0.04 [95% CI, –0.07 to –0.01]; *P* = .006). Adding quadratic terms revealed *U*-shaped associations for CV (CV^2^: β = –0.002 [95% CI, –0.003 to –0.001]; *P* < .001) and AUCg (AUCg^2^: β = 0.006 [95% CI, 0.00 to 0.01]; *P* = .08). Full results are presented in eTable 4 in Supplement 1.

### Cortisol Quintiles and Incident AD

In fully adjusted discrete-time survival analysis models with limited case counts in the smaller adjudicated subsample of 825 participants, none of the cortisol indices were significantly associated with incident AD, which was observed in 92 participants. Odds ratios across quintiles were close to null with wide 95% CIs, consistent with limited statistical power (eTable 5 in Supplement 1).

### Race as a Moderator of Cortisol-Cognition Associations

**Figure 4.**
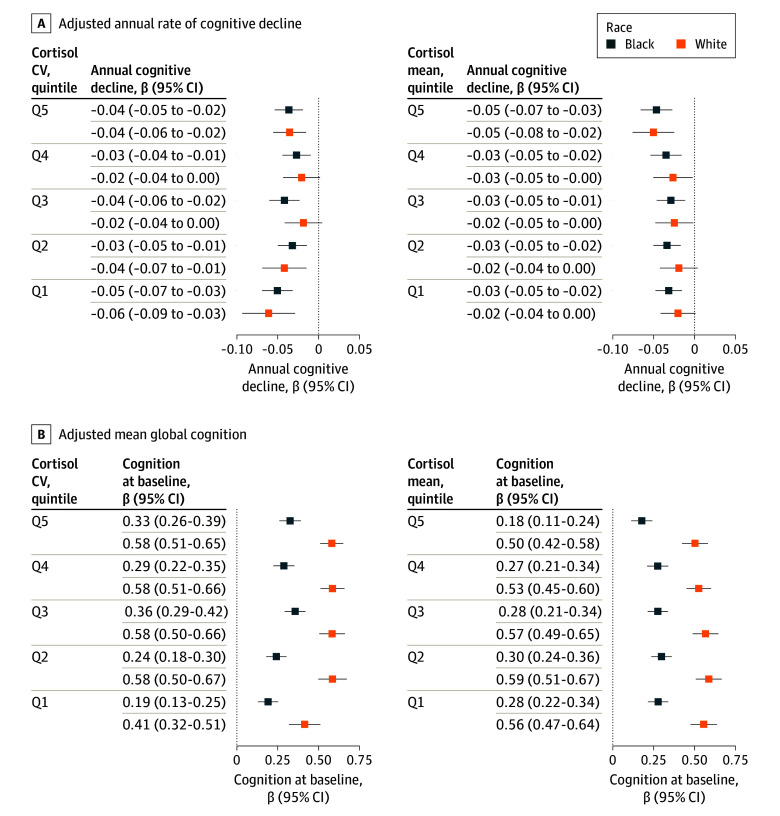
Dot Plots of Adjusted Mean Global Cognition at Baseline and Annual Rate of Cognitive Decline Stratified by Race β values are given as standardized units. A, Higher cumulative cortisol exposure was associated with poorer cognitive performance at the highest quintile (Q5) across racial groups. Patterns were similar by race, although distributions differed. B, Higher cumulative cortisol exposure was associated with faster cognitive decline at the highest quintile (Q5), with broadly similar associations across racial groups. Models adjusted for age, sex, race, education, body mass index, comorbidities, medication use, *APOE ε4 *status, smoking, and alcohol use.

No significant race × cortisol interactions were observed across indices in fully adjusted models (eTable 6 and eFigure 3 in Supplement 1; Figure 4A-B), indicating that the direction and magnitude of cortisol-cognition associations were broadly similar across racial groups. Stratified analyses showed consistent patterns across groups (eTable 6 in Supplement 1).

### Sensitivity Analyses

Results were similar in analyses excluding participants with baseline AD, with cross-sectional associations preserved across indices and a significant negative association observed for Q5 mean cortisol (β = −0.01 [95% CI −0.02 to −0.001]; *P* = .03). These findings indicate that results were not driven by participants with low baseline cognitive performance. Detailed results are presented in eTable 7 in Supplement 1.

## Discussion

In this cohort study examining salivary cortisol levels and cognitive decline, across the 5 salivary cortisol indices, distinct yet complementary patterns emerged that reflect distinct aspects of diurnal cortisol patterning and associations with cognitive decline. All indices were associated cross-sectionally with cognitive performance, indicating that multiple dimensions of HPA-axis alterations (ie, intraday variability [CV], cumulative [mean cortisol, AUCg], and diurnal change [slope, AUCi]) tracked with concurrent cognitive function. In longitudinal analyses, distinct associations emerged across cortisol indices, with moderate intraday variability associated with slower decline and higher cumulative exposure associated with faster decline in the full analytic sample. When participants with the lowest 10% of baseline cognitive performance were excluded, mean cortisol remained significant, underscoring its robustness as an early physiological marker associated with subsequent cognitive decline among cognitively healthy adults. Exploratory analyses showed that no cortisol index was associated with incident AD, likely reflecting limited case counts and shorter follow-up relative to the long preclinical phase of AD. Notably, Black participants exhibited lower cumulative cortisol exposure but flatter diurnal slopes and lower intraday variability, consistent with a blunted diurnal rhythm rather than hypersecretion. Nonetheless, the magnitude of associations with cognitive decline were similar across racial groups, suggesting that these diurnal cortisol patterns were associated with comparable cognitive outcomes across groups. Notably, to our knowledge, this is the first large racially diverse cohort study to show that although cortisol-cognition associations were comparable across racial groups, underlying cortisol profiles differed systematically, highlighting patterns consistent with theoretical frameworks in which social and structural adversity may be reflected in diurnal cortisol patterning and disproportionately burden Black adults, even without altering cortisol-cognition associations. Collectively, these findings position salivary cortisol as an early, physiologically relevant, and modifiable biomarker associated with stress-related neurocognitive aging, with implications for early prevention and health equity. Specifically, intraday variability and cumulative indices (CV, mean cortisol, AUCg) may inform longitudinal risk stratification in community settings, whereas diurnal change indices (slope, AUCi) are better suited for mechanistic or intervention studies targeting acute or short-term stress regulation.

### Physiological Interpretation of Individual Indices

Because each cortisol index reflects a distinct dimension of HPA-axis physiology, concurrent examination provides complementary insights into the mechanisms linking stress physiology to cognitive aging. The intraday variability index (CV) captures stability and daily fluctuations of cortisol output. It reflects the dispersion of cortisol values across sampled time points within a single day. In this study, CV was associated with both baseline cognition and longitudinal change, with an inverse *U*-shaped pattern, suggesting that moderate levels were associated with more favorable outcomes. Moderate CV reflects balanced intraday dispersion across sampled cortisol values, whereas excessive fluctuations in either direction may suggest impaired intraday feedback and high allostatic load. Correspondingly, in this study, CV was consistently associated with both baseline cognition and decline, with an inverse *U*-shaped pattern indicating that moderate to high variability was most closely associated with slower decline. These findings extend prior small-sample work in older adults^44^ and align with developmental work suggesting that both blunted and excessive variability can be detrimental.^45^

The cumulative exposure indices (mean cortisol and AUCg) capture day-level cortisol output, with mean cortisol indicating basal levels while AUCg emphasizes total daily exposure. Higher cortisol levels have been associated with hippocampal atrophy, memory deficits, and accelerated decline in prior studies.^32,46^ In this study, only the highest quintile of mean cortisol and AUCg was associated with faster decline, suggesting a threshold pattern: moderate levels may be neutral, but hypercortisolism at the extreme can be detrimental. Notably, few studies have evaluated AUCg in association with cognitive outcomes; one longitudinal study reported discordant associations,^17^ highlighting the influence of methodological and cohort differences, eg, outcome measurement, sample composition, or follow-up length.

The diurnal change indices (slope and AUCi) reflect directional changes in diurnal cortisol output. Flatter slopes were associated with contemporaneous cognition but not future decline, and AUCi, often used to index acute reactivity, showed similar associations limited to baseline cognition. This pattern suggests that these diurnal change indices reflect transient changes in diurnal cortisol patterns or daily routines rather than processes relevant to long-term cognitive trajectories. In the literature, longitudinal findings remain mixed, with some cohorts reporting flatter slopes as significant while others found null results.^47^ Methodological variation in sampling intensity, cohort composition (clinical vs community-based cohorts),^48^ and follow-up length likely contribute.

### Multidimensional HPA-Axis Patterning Across All Cortisol Indices

Taken together, our findings align with prior evidence that mean and AUCg cortisol reflect cumulative HPA-axis output and physiological burden,^25,26^ whereas diurnal change and intraday variability indices (slope, AUCi, CV) capture short-term regulatory processes that become more disrupted with emerging cognitive impairment.^15,24,30,49^ Consistent with these results, individuals with mild cognitive impairment or AD showed higher CSF cortisol levels.^15,50^ Similar to our findings comparing cortisol profiles between individuals who eventually developed AD vs those who did not, prior studies^31,47,48^ have shown that individuals with incident AD exhibit a blunted diurnal cortisol rhythm, characterized by lower intraday variability and a flatter diurnal slope, providing biological corroboration for our salivary findings. In all, across a series of robust longitudinal analyses, the salivary index reflecting cumulative cortisol burden emerged as the most stable and physiologically interpretable risk factor associated with of stress-related cognitive aging, underscoring its potential utility as an early biomarker of prospective cognitive decline.

### Incident AD

Null associations with incident AD likely reflect sharply reduced statistical power in the exploratory subsample (from nearly 4000 to approximately 800 participants, including only 92 participants with incident AD) and the long preclinical phase (up to 20 years) not captured in our shorter follow-up (up to 11 years). In contrast, cortisol indices were associated with cognitive decline in the much larger full cohort, suggesting that stress physiology functions as an earlier, more sensitive marker of subtle preclinical neurocognitive aging preceding AD. Taken together, salivary cortisol may have greater relevance for early identification of early cognitive decline than for shorter-term AD incidence, aligning with the principle that interventions are most effective before clinical AD emerges. These findings further support an X dimension in the ATX(N) framework,^4^ reflecting systemic processes, such as stress physiology, inflammation, and metabolic processes, that may contribute to early neurocognitive aging independent of amyloid or tau pathology.

### Racial Disparities

The racially diverse design of this cohort provided a unique opportunity to examine disparities in stress physiology with direct relevance for tailoring early biomarkers and prevention across diverse older populations. Distinct racial differences were observed in baseline cortisol profiles: Black participants exhibited lower cumulative cortisol exposure (mean and AUCg) yet less pronounced diurnal variation, characterized by flatter diurnal slopes and blunted AUCi and lower CV, reflecting a blunted rhythm rather than generalized hypersecretion. This distinct profile is consistent with theoretical frameworks suggesting that social and structural adversity may be reflected in diurnal cortisol patterning, producing attenuated daily amplitude as a physiological response to sustained stress,^51,52,53^ although such exposures were not directly measured in this study. Subsequently, for the associations with cognitive outcomes, although no significant race moderation was detected, cognitive decline was faster among Black than White participants, and the overall pattern indicated that differences in diurnal cortisol profiles, characterized by lower intraday variability and less pronounced diurnal variation cortisol profiles, were associated with accelerated cognitive aging. Despite baseline physiological differences, the associations between cortisol indices and cognitive decline were directionally similar, with no significant differences in effect sizes by race. These findings align with allostatic load and weathering frameworks, which posit that racial disparities in stress physiology may reflect disproportionate exposure to psychosocial stressors and their cumulative biological wear and tear effects in marginalized populations, rather than intrinsic biological vulnerability.^54,55^ Additional psychosocial processes, such as heightened vigilance, sleep disruption, and reduced restorative recovery, may collectively contribute to blunted diurnal cortisol patterns.^26,56,57,58^ Collectively, this biracial design provides objective physiological evidence of racial differences in diurnal cortisol patterns, with implications for equity-informed strategies to mitigate stress-related cognitive disparities.^59^

### Strengths and Limitations

This study has some strengths. Compared with CSF, blood, or urinary cortisol, salivary cortisol offers greater physiologic specificity and avoids acute stress responses to venipuncture.^13^ It provides a direct measure of biologically active, unbound hormone and therefore is less affected by comorbidities and cortisol-binding globulin, which varies by age and sex.^60,61,62^ This enhanced specificity may explain why some salivary-based indices in this study showed associations with cognition that differed from previous blood or urinary studies.^30^ Another distinct advantage of salivary cortisol is the possibility of repeated daily sampling that enabled derivation of 5 complementary, physiologically distinct indices, providing a multidimensional characterization of diurnal HPA-axis activity that extends beyond what can be captured from single CSF or blood sample. Sensitivity analyses excluding participants with the lowest baseline cognitive performance reduced concerns about reverse causation, and prospective modeling of within-person cognitive change strengthened temporal inference. In addition to prior work examining salivary cortisol and cognitive outcomes, this study extends the literature by jointly evaluating longitudinal cognitive decline and incident AD within the same framework. The study uniquely examined nonlinear cortisol-cognition associations and tracked both longitudinal cognitive decline and incident AD, addressing previous gaps. The study further demonstrated a threshold association, with participants having the highest quintile of cumulative cortisol exposure (mean cortisol) showing robust significant prospective cognitive decline. Notably, the large, community-based racially diverse cohort of nearly 4000 older adults, including more than 60% representation of Black older adults, represents 1 of the largest and most diverse population-based studies incorporating salivary cortisol sampling and longitudinal cognitive assessment in aging. The cohort enabled direct comparison between racial groups and substantially extends prior population-based studies by enhancing generalizability beyond prior clinic-based studies among predominantly White participants. These data provide rare population-based evidence on racial differences in stress physiology and cognitive aging. Adjustment for demographic, behavioral, and genetic covariates further improved internal validity.^30^

This study also has some limitations. One limitation is that the 3-sample protocol did not permit estimation of the cortisol awakening response. However, expert consensus indicates that 3 daily samples provide adequate characterization of the diurnal rhythm,^30^ and the cortisol awakening response represents a distinct physiological process separate from the remainder of the diurnal profile.^23,24^ Although multiday sampling may improve reliability of within-person variability and longer-term HPA-axis dynamics, particularly in smaller or intensive sampling studies,^29,63^ single-day multi–time point protocols remain commonly used in large epidemiologic studies as a pragmatic and scalable approach to capture diurnal cortisol features at the population level while minimizing participant burden.^17,24,30,31,32^ Accordingly, CV derived from 3 within-day samples should be interpreted as an index of dispersion across the sampled diurnal profile rather than as a measure of stable day-to-day variability or longer-term HPA-axis regulation. These approaches capture related but distinct physiological features, and denser multiday sampling may provide complementary information regarding longer-term within-person cortisol dynamics. Perceived stress was not assessed alongside cortisol; these constructs reflect related but distinct dimensions of stress, and their joint evaluation represents an important direction for future research.^64^ As an observational study, residual confounding and reverse causation cannot be completely excluded, despite adjustment for demographic, clinical, and behavioral factors and sensitivity analyses excluding participants with low baseline cognition. For instance, kidney function measures were not available in this cohort, although kidney dysfunction may more substantially influence circulating blood-based cortisol measures through altered metabolism and clearance. Furthermore, results were robust after excluding participants with the lowest baseline global cognition, consistent with our prior analyses.^41,42,43,65^ As the CHAP study started in 1993, AD diagnoses were based on National Institute of Neurological and Communicative Disorders and Stroke and the Alzheimer Disease and Related Disorders Association criteria, which were the criterion standard at the time and which has yet to incorporate contemporary biomarker-informed frameworks. Additionally, the comparatively smaller number of adjudicated AD cases and limited follow-up may have reduced statistical power to detect associations with incident AD, rather than the absence of a biological relationship.

## Conclusions

In this cohort study of 3895 Black and White older adults, altered diurnal cortisol patterning and higher cumulative cortisol exposure were associated with faster longitudinal cognitive decline but not short-term AD risk. When combined with other cumulative and intraday variability indices, these measures inform longitudinal risk factors in cognitively mixed community samples. In contrast, diurnal change indices appear to capture short-term stress physiology more relevant to acute stress paradigms and intervention design than to long-term cognitive trajectories. Importantly, distinct cortisol profiles among Black participants, marked by flatter diurnal slopes and lower intraday variability, are consistent with theoretical frameworks suggesting that differences in exposure to social and structural stressors may recalibrate baseline stress physiology without necessarily altering cortisol-cognition associations. Because cortisol patterns may be responsive to behavioral, pharmacologic, and social interventions, these findings underscore opportunities for precision prevention and equity-informed strategies targeting stress-related cognitive aging.

## Notes

Supplement 1. eMethods. Salivary Cortisol Indices and Derivation eTable 1. Salivary Cortisol Indices: Definitions, Physiological and Analytic Interpretation eFigure 1. Flow of Participants in the CHAP Salivary Cortisol Study eFigure 2. Associations of Diurnal Dynamic Cortisol Indices With Cognitive Outcomes eFigure 3. Race-Stratified Associations of Cortisol Indices With Cognitive Outcomes eTable 2. Participant Characteristics by Incident Alzheimer Disease Status eTable 3. Associations of Cortisol Indices With Global Cognition (Quintile Models) eTable 4. Associations of Continuous Cortisol Indices With Global Cognition eTable 5. Associations of Cortisol Indices With Incident Alzheimer Disease eTable 6. Race Interaction and Stratified Models eTable 7. Sensitivity Analyses

Supplement 2. Data Sharing Statement
