# Supplementary material for: Salivary Cortisol and Cognitive Decline and Alzheimer Disease in Older Adults
Source: JAMA Netw Open. 2026 Jul 15;9(7):e2622955. doi: 10.1001/jamanetworkopen.2026.22955 (PMC13373666; doi:10.1001/jamanetworkopen.2026.22955)
Supplement: Supplement 1. — eMethods. Salivary Cortisol Indices and Derivation eTable 1. Salivary Cortisol Indices: Definitions, Physiological and Analytic Interpretation eFigure 1. Flow of Participants in the CHAP Salivary Cortisol Study eFigure 2. Associations of Diurnal Dynamic Cortisol Indices With Cognitive Outcomes eFigure 3. Race-Stratified Associations of Cortisol Indices With Cognitive Outcomes eTable 2. Participant Characteristics by Incident Alzheimer Disease Status eTable 3. Associations of Cortisol Indices With Global Cognition (Quintile Models) eTable 4. Associations of Continuous Cortisol Indices With Global Cognition eTable 5. Associations of Cortisol Indices With Incident Alzheimer Disease eTable 6. Race Interaction and Stratified Models eTable 7. Sensitivity Analyses [file jamanetwopen-e2622955-s001.pdf]

Supplemental Online Content

Ng TS, Beck T, Sudhini Y, Wilson RS, Evans DA, Rajan KB. Salivary cortisol and cognitive decline and Alzheimer disease in older adults. *JAMA Netw Open*. 2026;9(7):e2622955.
doi:10.1001/jamanetworkopen.2026.22955

eMethods. Salivary Cortisol Indices and Derivation

eTable 1. Salivary Cortisol Indices: Definitions, Physiological and Analytic Interpretation

eFigure 1. Flow of Participants in the CHAP Salivary Cortisol Study

eFigure 2. Associations of Diurnal Dynamic Cortisol Indices with Cognitive Outcomes

eFigure 3. Race-Stratified Associations of Cortisol Indices with Cognitive Outcomes

eTable 2. Participant Characteristics by Incident Alzheimer Disease Status

eTable 3. Associations of Cortisol Indices with Global Cognition (Quintile Models)

eTable 4. Associations of Continuous Cortisol Indices with Global Cognition

eTable 5. Associations of Cortisol Indices with Incident Alzheimer Disease

eTable 6. Race Interaction and Stratified Models

eTable 7. Sensitivity Analyses

This supplemental material has been provided by the authors to give readers additional information about their work.

© 2026 Ng TS et al. *JAMA Network Open*.

eMethods. Salivary Cortisol Indices and Derivation

Dysregulated diurnal cortisol rhythms can be indexed in several ways, each capturing a distinct physiological role. In AD research, single time-point levels or morning-to-evening ratios are most common, whereas broader psychoneuroendocrinological studies employing multiple daily salivary samples frequently examine (a) variability across the day (CV), (b) cumulative exposure (mean cortisol and AUCg), and (c) dynamic indices (diurnal slope and AUCi), which we will focus in this study. Each of these indices has a distinct physiological meaning (**eTable 1, Figure 1**).

For this study, we prespecified one primary, two secondary, and two exploratory indices, guided by prior literature and biological plausibility. Based on the diurnal curve (**Figure 1**), we derived five complementary indices of salivary cortisol to capture distinct dimensions of hypothalamic–pituitary–adrenal (HPA) axis activity: the primary index was variability (CV); the secondary indices were cumulative exposure (Mean Cortisol and AUCg); and the exploratory dynamic indices were diurnal slope and AUCi. Their definitions, physiological interpretations, and analytic roles are summarized in **eTable 1**, with a schematic illustration provided in **Figure 1**.

Primary Index: Intra-day Variability (Coefficient of Variation, CV).

CV captures within-day variability in cortisol output, reflecting the stability and adaptability of HPA axis regulation. Variability has been linked to stress responsivity and cognitive performance across developmental and aging cohorts. Given its sensitivity to both hypo- and hyperactivation (inverse U-shaped associations), CV was designated the primary index.

Secondary Indices: Cumulative Exposure (Mean Cortisol and AUCg).

Mean cortisol emphasizes overall basal levels but is highly correlated with AUCg. The area under the curve with respect to ground (AUCg) reflects cumulative daily cortisol burden, integrating basal output across the day, and is a standard index in psychoneuroendocrinology. Specifically, AUCg represents the total area under the cortisol curve relative to zero (i.e., cumulative cortisol exposure across the day). They were therefore designated as secondary indices.

Exploratory Indices: Diurnal change (Diurnal Slope and AUCi).

The diurnal slope captures the rate of decline across the day and has been associated with stress, sleep, and health outcomes; the area under the curve with respect to increase (AUCi) quantifies diurnal change relative to baseline and is often used in *acute* stress paradigms. However, prior findings on its cognitive relevance are relatively scarce and inconsistent. Accordingly, these indices were considered exploratory.

© 2026 Ng TS et al. *JAMA Network Open*.

eTable 1. Salivary Cortisol Indices: Definitions, Physiological & Statistical Interpretation, and Analytic Role

Index	Definition / Formula	Physiological interpretation	Statistical / Analytic interpretation	Role	Rationale
Coefficient of variation (CV)	SD of 3 cortisol values ÷ mean cortisol	Reflects HPA axis stability and adaptability; too low = rigid/blunted; too high = erratic regulation and wear-and-tear/high allostatic load	Captures intra-day variability; sensitive to non-linear (e.g.,inverse U-shaped) associations	Primary	Robust variability index with prior links to cognitive outcomes
Mean cortisol	$(C_1 + C_2 + C_3)/3$	Represents average daily cortisol level (basal exposure)	Summarizes overall level; highly correlated with AUCg; emphasizes basal level not rhythm	Secondary	Provides a simple, interpretable measure of basal cortisol level. Similar yet distinct to AUCg; mean cortisol captures basal level while AUCg indicates total exposure/stress burden
AUCg (area under the curve, ground)	Trapezoidal method: $[(C1 + C2)/2 \times t2-t1] + [(C2 + C3)/2 \times t3-t2]$	Integrates total daily cortisol exposure (cumulative HPA-axis output; allostatic load)	Captures chronic HPA overactivation (high) or underactivation (low)	Secondary	Standard index in psychoneuroendocrine studies; reflects cumulative stress exposure
Diurnal slope	Regression of log-transformed cortisol	Reflects circadian decline	Captures rate of decline across the	Exploratory	Mixed prior findings; cross-sectional relevance

© 2026 Ng TS et al. *JAMA Network Open*.

	values on time of day (t_1–t_3)	('shut-off' efficiency); flatter slopes indicate dysregulation	day; sensitive to timing and spacing of samples. Steeper = efficient regulation; flatter = stress, aging, depression		more than longitudinal
AUCi (area under the curve, increase)	Trapezoidal method using change from baseline (morning): ((C2−C1)+0)/2×(t2−t1)($(C_2−C_1) + 0$) / 2 × $(t_2−t_1)$((C2−C1)+0)/2×(t2−t1) + ((C3−C1)+(C2−C1))/2×(t3−t2)($(C_3−C_1) + (C_2−C_1)$) / 2 × $(t_3−t_2)$((C3−C1)+(C2−C1))/2×(t3−t2)	Captures diurnal change relative to baseline; reflects HPA-axis adaptability and reactivity	Quantifies deviation from baseline across the day; distinguishes increase vs decline patterns independent of absolute level	Exploratory	Complements AUCg by isolating change rather than total exposure; widely used in *acute* stress paradigms; less established for cognition and aging

Notes: C_1, C_2, and C_3 denote cortisol concentrations at the first (waking), second (afternoon), and third (bedtime) samples, respectively. t_1, t_2, and t_3 denote the corresponding sampling times. AUCi was calculated using the trapezoidal method based on change from baseline (C_1).

© 2026 Ng TS et al. *JAMA Network Open*.

eFigure 1. Flow of Participants in the Chicago Health and Aging Project Salivary Cortisol Study

Flow of participants included in salivary cortisol and cognitive analyses in the Chicago Health and Aging Project (CHAP). Of 5008 eligible individuals, 4618 accepted saliva collection kits, and 3898 returned at least 1 valid cortisol sample. After excluding 3 participants with missing education data, 3895 were included in the primary cortisol–cognition analyses. A subsample of 825 participants had clinical evaluations for incident Alzheimer's disease.

eFigure 1. Flow Diagram of Participants

5008 Eligible for Salivary Cortisol Collection

↓

4618 Accepted Saliva Collection Tubes

↓

3898 Returned ≥1 Valid Cortisol Sample

↓

3895 Included in Cortisol–Cognition Analyses
(3 excluded due to missing education)

↓

825 Subsample With Clinical Evaluation for Incident Alzheimer Disease

Flow of participants included in salivary cortisol and cognitive analyses in the Chicago Health and Aging Project (CHAP). Of 5008 eligible individuals, 4618 accepted saliva collection kits and 3898 returned at least one valid cortisol sample. After excluding 3 individuals missing education data, 3895 were included in the primary cortisol–cognition models. A subset of 825 participants had clinical evaluations available for incident Alzheimer disease analyses.

© 2026 Ng TS et al. *JAMA Network Open*.

eFigure 2. Associations of salivary cortisol diurnal change indices with cognitive outcomes.

eFigure 2a. Adjusted mean global cognition at baseline across quintiles (Q1–Q5) of salivary cortisol indices (diurnal slope, and area under the curve with respect to increase [AUCi]).

Diurnal dynamic indices (slope and AUCi) showed weaker and less consistent associations with baseline cognition.

Estimates are presented as β coefficients with 95% CIs in a forest plot format, with numerical values displayed alongside graphical elements. Models were adjusted for age, sex, race, education, BMI, comorbidities, medication use, APOE ε4 status, smoking, and alcohol use. Asterisks indicate P<.05 vs Q1 (reference).

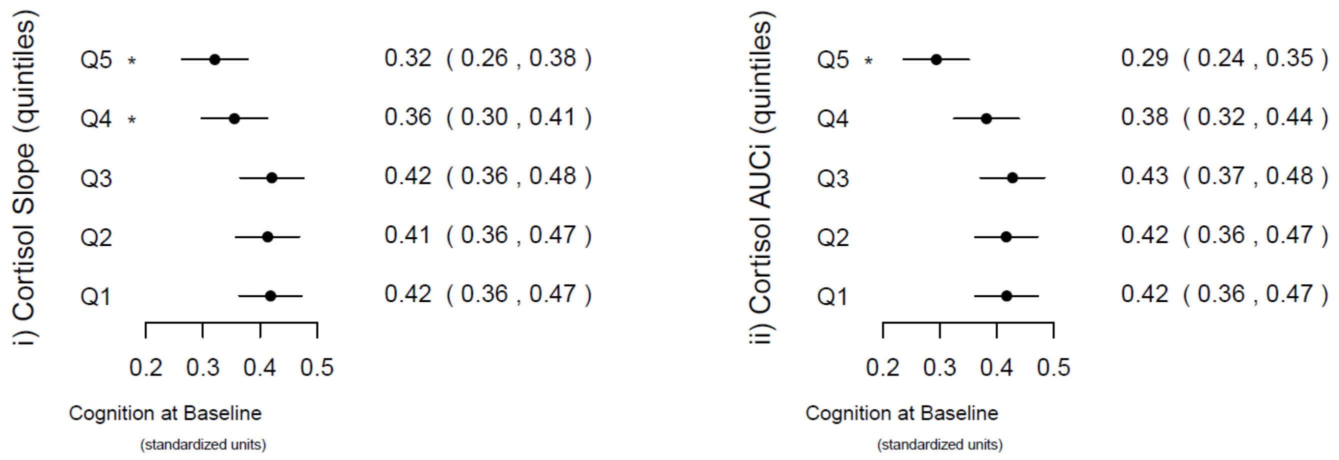

© 2026 Ng TS et al. *JAMA Network Open*.

eFigure 2b. Adjusted annual rate of cognitive decline across quintiles (Q1–Q5) of salivary cortisol indices (diurnal slope, and AUCi).

Diurnal dynamic indices (slope and AUCi) were not significantly associated with longitudinal change.

Estimates are presented as β coefficients with 95% CIs in a forest plot format, with numerical values displayed alongside graphical elements. Models were adjusted. Asterisks indicate P<.05 vs Q1.

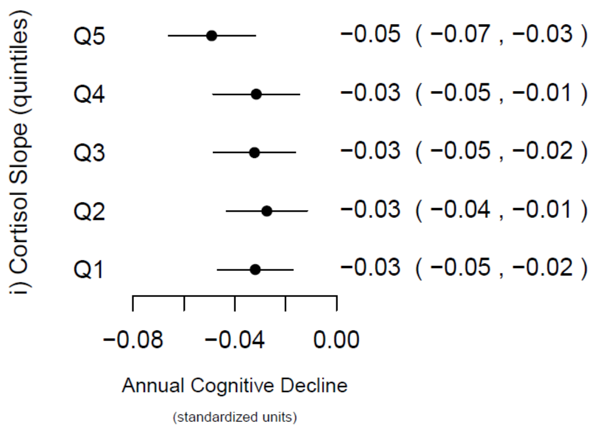

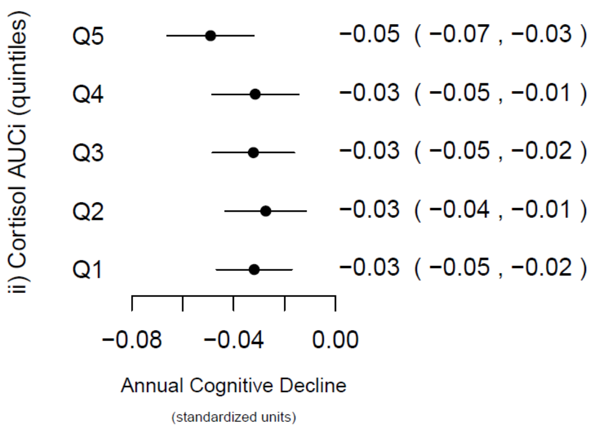

© 2026 Ng TS et al. *JAMA Network Open*.

eFigure 3. Associations of diurnal dynamic salivary cortisol indices with cognitive outcomes.

eFigure 3a. Associations of Diurnal Cortisol Measures With Annual Cognitive Decline, by Race

Estimates represent β coefficients (95% CIs) for annual change in global cognition (standardized units) across quintiles of diurnal cortisol measures, with the lowest quintile (Q1) as the reference.
Panels show (i) area under the curve with respect to ground (AUCg), (ii) diurnal slope, and (iii) area under the curve with respect to increase (AUCi).

Models were adjusted for age, sex, education, body mass index, medical comorbidities, APOE ε4 status, medication use (antidepressants, steroids), alcohol use, and smoking.

Results are stratified by race (Black and White participants). Negative values indicate faster cognitive decline.

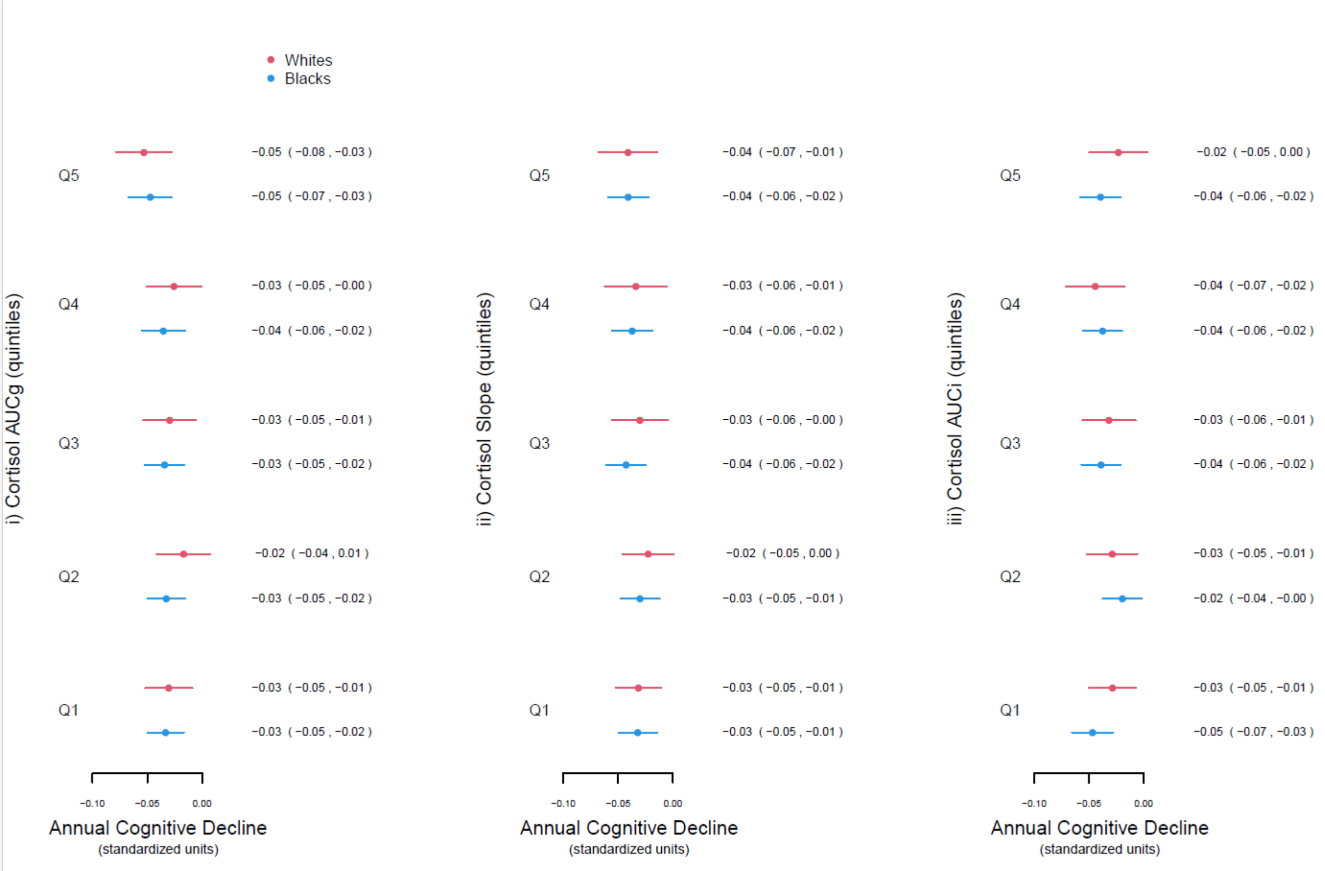

© 2026 Ng TS et al. *JAMA Network Open*.

eFigure 3B. Associations of Diurnal Cortisol Measures With Baseline Global Cognition, by Race

Estimates represent adjusted mean differences (95% CIs) in baseline global cognition (standardized units) across quintiles of diurnal cortisol measures, with the lowest quintile (Q1) as the reference.
Panels show (i) area under the curve with respect to ground (AUCg), (ii) diurnal slope, and (iii) area under the curve with respect to increase (AUCi).

Models were adjusted for age, sex, education, body mass index, medical comorbidities, APOE ε4 status, medication use (antidepressants, steroids), alcohol use, and smoking.

Results are stratified by race (Black and White participants). Lower values indicate poorer cognitive performance.

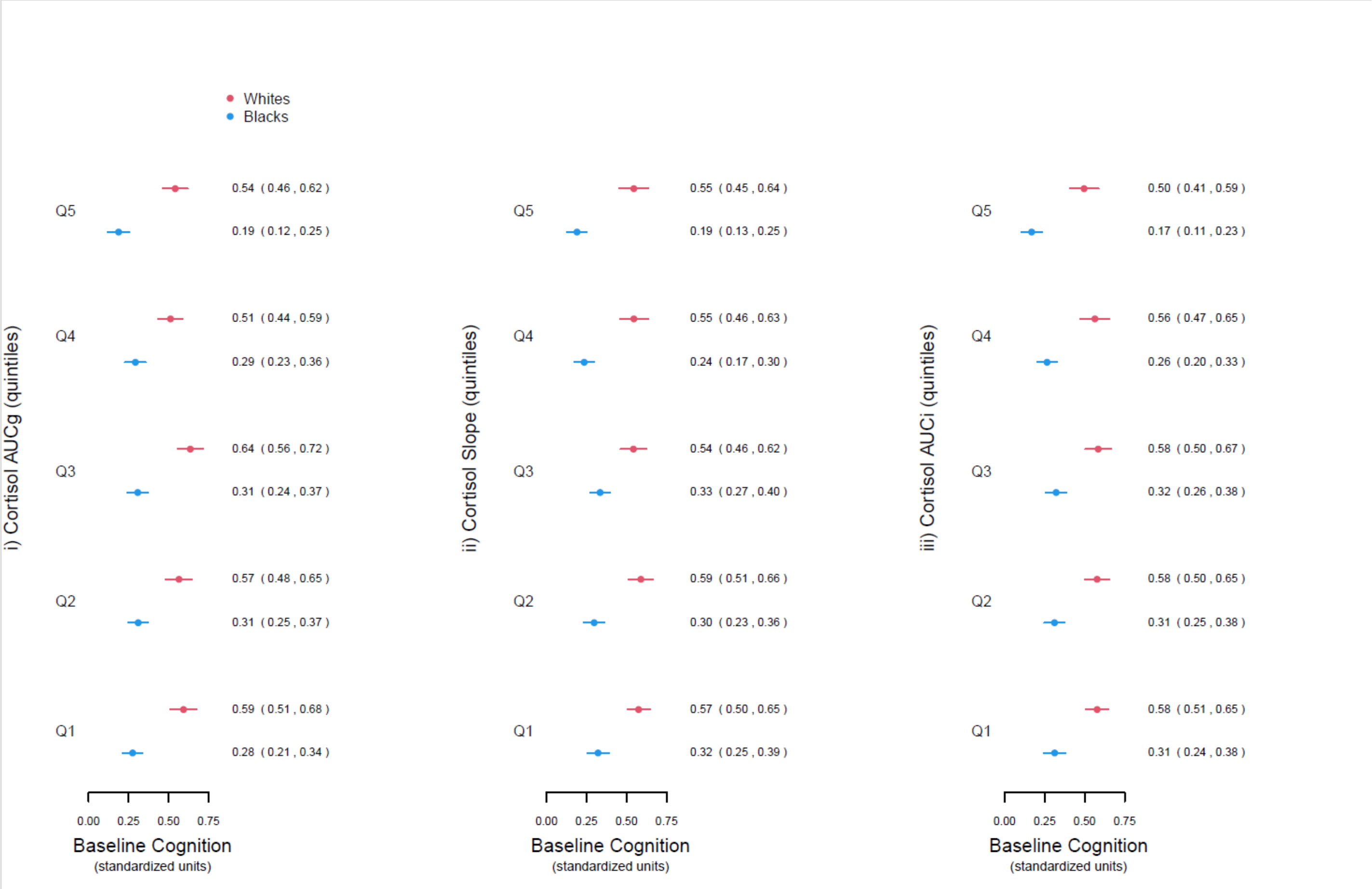

© 2026 Ng TS et al. *JAMA Network Open*.

eTable 2. Demographics of Participants with and without Incident AD at follow-up.

	Overall<br>N = 825[1]	Non-AD<br>N=733[1]	AD<br>N=92[1]	p-value[2]
Age, years	78.9 (6.4)	78.6 (6.4)	81.3 (6.0)	<0.001*
Female	515 (62%)	456 (62%)	59 (64%)	0.81
Black	499 (60%)	429 (59%)	70 (76%)	0.002*
Education, years	13.0 (3.3)	13.2 (3.3)	12.0 (3.3)	0.002*
Cortisol CV	74.6 (35.2)	75.5 (35.3)	67.2 (33.6)	0.030*
Mean Cortisol	7.3 (5.0, 10.7)	7.2 (5.0, 10.7)	7.3 (5.3, 11.2)	0.65
AUCg	7.7 (5.3, 11.1)	7.7 (5.2, 11.0)	7.8 (5.4, 11.7)	0.46
Slope of Cortisol	-0.074 (0.030)	-0.075 (0.030)	-0.068 (0.027)	0.042*
AUCi	-5.1 (-10.0, -1.1)	-5.3 (-10.2, -1.3)	-4.1 (-8.0, -0.4)	0.046*
BMI	27.5 (5.7)	27.7 (5.8)	26.2 (4.7)	0.006*
Medical Conditions	1.52 (0.98)	1.50 (0.97)	1.62 (1.03)	0.31
Anti-depressant	53 (6.4%)	48 (6.5%)	5 (5.4%)	0.85
Steroids	38 (4.6%)	33 (4.5%)	5 (5.4%)	0.89
Alcohol - Moderate	237 (29%)	217 (30%)	20 (22%)	0.15
Alcohol - High	46 (5.6%)	46 (6.3%)	0 (0%)	0.026*
APOE-4 Carrier	235 (29%)	201 (28%)	34 (37%)	0.078

[1]Mean (SD); n (%); Median (IQR)

[2]Welch Two Sample t-test; Pearson's Chi-squared test; Wilcoxon rank sum test

Abbreviations: AUCg (area under the curve, ground); AUCi (area under the curve, increase); BMI=body-mass index; * indicates p <0.05.

© 2026 Ng TS et al. *JAMA Network Open*.

eTable 3a. Cross-sectional associations of baseline cortisol indices with global cognition (linear mixed-effects models)

Primary Index: Coefficient of variation (CV)

Quintile	Model 1 β (95% CI)	p	Model 2 β (95% CI)	p	Model 3 β (95% CI)	p
Q1	Reference	Reference	Reference	Reference	Reference	Reference
**Q2**	**0.10 (0.04–0.16)**	**0.001**	**0.08 (0.02–0.14)**	**0.01**	**0.08 (0.03–0.14)**	**0.005**
**Q3**	**0.19 (0.13–0.25)**	**0.000**	**0.16 (0.10–0.22)**	**0.000**	**0.16 (0.10–0.22)**	**0.000**
**Q4**	**0.16 (0.10–0.22)**	**0.000**	**0.12 (0.07–0.18)**	**0.000**	**0.12 (0.06–0.18)**	**0.000**
**Q5**	**0.16 (0.10–0.22)**	**0.000**	**0.14 (0.08–0.20)**	**0.000**	**0.14 (0.08–0.20)**	**0.000**

n = Model 1: 3711; Model 2: 3338; Model 3: 3337

Secondary Index: Mean cortisol

Quintile	Model 1 β (95% CI)	p	Model 2 β (95% CI)	p	Model 3 β (95% CI)	p
Q1	Reference	Reference	Reference	Reference	Reference	Reference
Q2	0.03 (-0.02–0.09)	0.24	0.03 (-0.03–0.09)	0.33	0.02 (-0.03–0.08)	0.40
Q3	0.01 (-0.04–0.07)	0.63	0.01 (-0.05–0.07)	0.77	0.003 (-0.05–0.06)	0.93
Q4	-0.01 (-0.07–0.05)	0.74	-0.01 (-0.07–0.05)	0.69	-0.01 (-0.07–0.04)	0.62
**Q5**	**-0.11 (-0.17–-0.05)**	**0.000**	**-0.08 (-0.14–-0.02)**	**0.01**	**-0.08 (-0.14–-0.02)**	**0.01**

n = Model 1: 3895; Model 2: 3496; Model 3: 3495

Secondary Index: AUCg

© 2026 Ng TS et al. *JAMA Network Open*.

Quintile	Model 1 β (95% CI)	p	Model 2 β (95% CI)	p	Model 3 β (95% CI)	p
Q1	Reference	Reference	Reference	Reference	Reference	Reference
Q2	0.02 (-0.04–0.08)	0.46	0.02 (-0.04–0.07)	0.58	0.01 (-0.04–0.07)	0.63
Q3	0.04 (-0.02–0.10)	0.20	0.04 (-0.02–0.10)	0.19	0.04 (-0.02–0.10)	0.21
Q4	-0.03 (-0.08–0.03)	0.40	-0.02 (-0.08–0.04)	0.51	-0.02 (-0.08–0.04)	0.46
**Q5**	**-0.10 (-0.16–-0.04)**	**0.001**	**-0.07 (-0.13–-0.01)**	**0.02**	**-0.07 (-0.13–-0.01)**	**0.02**

n = Model 1: 3618; Model 2: 3250; Model 3: 3249

Exploratory Index: Diurnal slope

Quintile	Model 1 β (95% CI)	p	Model 2 β (95% CI)	p	Model 3 β (95% CI)	p
Q1	Reference	Reference	Reference	Reference	Reference	Reference
Q2	-0.004 (-0.06–0.05)	0.88	-0.01 (-0.07–0.05)	0.73	-0.01 (-0.06–0.05)	0.86
Q3	-0.03 (-0.09–0.03)	0.39	-0.002 (-0.06–0.06)	0.96	0.002 (-0.06–0.06)	0.94
**Q4**	**-0.08 (-0.14–-0.02)**	**0.01**	**-0.07 (-0.13–-0.01)**	**0.02**	**-0.06 (-0.12–-0.005)**	**0.03**
**Q5**	**-0.12 (-0.18–-0.06)**	**0.000**	**-0.10 (-0.16–-0.04)**	**0.001**	**-0.10 (-0.16–-0.04)**	**0.001**

n = Model 1: 3618; Model 2: 3250; Model 3: 3249

Exploratory Index: AUCi

© 2026 Ng TS et al. *JAMA Network Open*.

Quintile	Model 1<br>β (95% CI)	p	Model 2<br>β (95% CI)	p	Model 3<br>β (95% CI)	p
Q1	Reference	Reference	Reference	Reference	Reference	Reference
Q2	0.02 (-0.04–0.08)	0.49	-0.002 (-0.06–0.06)	0.95	-0.001 (-0.06–0.06)	0.98
Q3	0.01 (-0.05–0.07)	0.82	0.01 (-0.05–0.06)	0.83	0.01 (-0.05–0.07)	0.73
Q4	-0.04 (-0.10–0.01)	0.14	-0.04 (-0.10–0.02)	0.19	-0.04 (-0.09–0.02)	0.23
**Q5**	**-0.14 (-0.20–-0.08)**	**0.000**	**-0.13 (-0.19–-0.07)**	**0.000**	**-0.12 (-0.18–-0.06)**	**0.000**

n = Model 1: 3618; Model 2: 3250; Model 3: 3249

Notes: Model 1 controlled for demographics (age, sex, education, and race). Model 2 additionally included health factors (chronic conditions, BMI, APOE ε4, antidepressant and glucocorticoid use). Model 3 further added health behaviors (smoking, alcohol use).

© 2026 Ng TS et al. *JAMA Network Open*.

eTable 3b. Longitudinal associations of baseline cortisol indices with rate of cognitive decline (linear mixed-effects models)

Primary Index: Coefficient of variation (CV)

Quintile	Model 1 β (95% CI)	p	Model 2 β (95% CI)	p	Model 3 β (95% CI)	p
Q1	Reference	Reference	Reference	Reference	Reference	Reference
**Q2**	0.02 (-0.002–0.04)	0.07	**0.02 (0.000–0.04)**	**0.04**	0.02 (-0.001–0.04)	0.06
**Q3**	**0.02 (-0.000–0.04)**	**0.05**	**0.02 (0.001–0.04)**	**0.04**	**0.02 (0.001–0.04)**	**0.04**
**Q4**	**0.03 (0.01–0.04)**	**0.01**	**0.03 (0.01–0.04)**	**0.003**	**0.03 (0.01–0.04)**	**0.003**
Q5	0.02 (0.003–0.04)	0.02	0.01 (-0.003–0.03)	0.11	0.02 (-0.002–0.03)	0.08

n = Model 1: 3711; Model 2: 3338; Model 3: 3337

Secondary Index: Mean cortisol

Quintile	Model 1 β (95% CI)	p	Model 2 β (95% CI)	p	Model 3 β (95% CI)	p
Q1	Reference	Reference	Reference	Reference	Reference	Reference
Q2	-0.003 (-0.02–0.01)	0.69	-0.000 (-0.02–0.01)	0.96	-0.001 (-0.02–0.01)	0.88
Q3	-0.004 (-0.02–0.01)	0.60	-0.001 (-0.02–0.01)	0.89	0.000 (-0.01–0.02)	0.96
Q4	-0.01 (-0.02–0.01)	0.36	-0.003 (-0.02–0.01)	0.68	-0.004 (-0.02–0.01)	0.62
**Q5**	**-0.01 (-0.03–0.002)**	**0.10**	**-0.02 (-0.04–-0.004)**	**0.01**	**-0.02 (-0.04–-0.004)**	**0.02**

n = Model 1: 3895; Model 2: 3496; Model 3: 3495

© 2026 Ng TS et al. *JAMA Network Open*.

Secondary Index: AUCg

Quintile	Model 1 β (95% CI)	p	Model 2 β (95% CI)	p	Model 3 β (95% CI)	p
Q1	Reference	Reference	Reference	Reference	Reference	Reference
Q2	0.001 (-0.01–0.02)	0.87	0.01 (-0.01–0.02)	0.49	0.004 (-0.01–0.02)	0.56
Q3	-0.003 (-0.02–0.01)	0.74	-0.000 (-0.02–0.02)	0.97	-0.000 (-0.02–0.02)	0.97
Q4	-0.005 (-0.02–0.01)	0.58	-0.001 (-0.02–0.02)	0.93	0.000 (-0.02–0.02)	0.96
**Q5**	**-0.01 (-0.03–0.01)**	**0.17**	**-0.02 (-0.03–-0.000)**	**0.049**	**-0.02 (-0.03–-0.000)**	**0.046**

n = Model 1: 3618; Model 2: 3250; Model 3: 3249

Exploratory Index: Diurnal slope

Quintile	Model 1 β (95% CI)	p	Model 2 β (95% CI)	p	Model 3 β (95% CI)	p
Q1	Reference	Reference	Reference	Reference	Reference	Reference
Q2	-0.001 (-0.02–0.02)	0.92	0.01 (-0.01–0.02)	0.52	0.005 (-0.01–0.02)	0.55
Q3	-0.01 (-0.03–0.01)	0.30	-0.01 (-0.02–0.01)	0.49	-0.01 (-0.02–0.01)	0.45
Q4	-0.01 (-0.02–0.01)	0.40	-0.004 (-0.02–0.01)	0.62	-0.004 (-0.02–0.01)	0.66
Q5	-0.02 (-0.03–0.000)	0.05	-0.01 (-0.03–0.01)	0.25	-0.01 (-0.02–0.01)	0.33

n = Model 1: 3618; Model 2: 3250; Model 3: 3249

Exploratory Index: AUCi

© 2026 Ng TS et al. *JAMA Network Open*.

Quintile	Model 1<br>β (95% CI)	p	Model 2<br>β (95% CI)	p	Model 3<br>β (95% CI)	p
Q1	Reference	Reference	Reference	Reference	Reference	Reference
Q2	0.01 (-0.01–0.03)	0.21	**0.02 (-0.000–0.03)**	**0.05**	0.02 (-0.001–0.03)	0.06
Q3	-0.01 (-0.02–0.01)	0.53	0.003 (-0.01–0.02)	0.70	0.003 (-0.01–0.02)	0.75
Q4	-0.01 (-0.02–0.01)	0.56	0.001 (-0.02–0.02)	0.90	0.001 (-0.02–0.02)	0.95
Q5	-0.003 (-0.02–0.01)	0.78	0.004 (-0.01–0.02)	0.69	0.005 (-0.01–0.02)	0.60

n = Model 1: 3618; Model 2: 3250; Model 3: 3249

Notes: Model 1 controlled for demographics (age, sex, education, and race). Model 2 additionally included health factors (chronic conditions, BMI, APOE ε4, antidepressant and glucocorticoid use). Model 3 further added health behaviors (smoking, alcohol use). Significant results ($p < 0.05$) are bolded.

Abbreviations: AUCg (area under the curve, ground); AUCi (area under the curve, increase)

© 2026 Ng TS et al. *JAMA Network Open*.

eTable 4a. Cross-sectional associations of continuous cortisol indices with global cognition — Linear-only models

Predictor	Model 1 β (95% CI)	p	Model 2 β (95% CI)	p	Model 3 β (95% CI)	p
Coefficient of variation (CV)	**0.02 (0.01, 0.02)**	**<0.001**	**0.01 (0.01, 0.02)**	**<0.001**	**0.01 (0.01, 0.02)**	**<0.001**

n = Model 1: 3711; Model 2: 3338; Model 3: 3337

Predictor	Model 1 β (95% CI)	p	Model 2 β (95% CI)	p	Model 3 β (95% CI)	p
Mean cortisol	-<0.0019 (-<0.001, -<0.001)	0.038	-<0.001 (-<0.001, <0.001)	0.14	-<0.001 (-<0.001, <0.001)	0.15

n = Model 1: 3895; Model 2: 3496; Model 3: 3495

Predictor	Model 1 β (95% CI)	p	Model 2 β (95% CI)	p	Model 3 β (95% CI)	p
AUCg	-0.0016 (-<0.001, -<0.001)	0.010	-<0.001 (-<0.001, <0.001)	0.15	-<0.001 (-<0.001, <0.001)	0.16

n = Model 1: 3618; Model 2: 3250; Model 3: 3249

Predictor	Model 1 β (95% CI)	p	Model 2 β (95% CI)	p	Model 3 β (95% CI)	p
AUCi (total change)	<0.001 (-<0.001, <0.001)	0.99	<0.001 (-<0.001, <0.001)	0.93	<0.001 (-<0.001, <0.001)	0.76

n = Model 1: 3618; Model 2: 3250; Model 3: 3249

Predictor	Model 1 β (95% CI)	p	Model 2 β (95% CI)	p	Model 3 β (95% CI)	p
Diurnal slope	**-1.29 (-1.96, -0.61)**	**<0.001**	**-1.15 (-1.82, -0.48)**	**<0.001**	**-1.10 (-1.77, -0.43)**	**0.001**

n = Model 1: 3618; Model 2: 3250; Model 3: 3249

© 2026 Ng TS et al. *JAMA Network Open*.

Notes: Model 1 controlled for demographics (age, sex, education, and race). Model 2 additionally included health factors (chronic conditions, BMI, APOE ε4, antidepressant and glucocorticoid use). Model 3 further added health behaviors (smoking, alcohol use). Significant results ($p < 0.05$) are bolded.

Abbreviations: AUCg (area under the curve, ground); AUCi (area under the curve, increase)

© 2026 Ng TS et al. *JAMA Network Open*.

eTable 4b. Longitudinal associations of continuous cortisol indices with global cognition — Linear-only models

Predictor	Model 1 β (95% CI)	p	Model 2 β (95% CI)	p	Model 3 β (95% CI)	p
Coefficient of variation (CV)	**0.002 (<0.001, <0.001)**	**0.025**	<0.001 (-<0.001, <0.001)	0.12	0.0014 (-<0.001, <0.001)	0.08

n = Model 1: 3711; Model 2: 3338; Model 3: 3337

Predictor	Model 1 β (95% CI)	p	Model 2 β (95% CI)	p	Model 3 β (95% CI)	p
Mean cortisol	-<0.0012 (-<0.001, -<0.001)	0.24	**-<0.0013 (-<0.001, -<0.001)**	**0.041**	**-<0.0013 (-<0.001, -<0.001)**	**0.044**

n = Model 1: 3895; Model 2: 3496; Model 3: 3495

Predictor	Model 1 β (95% CI)	p	Model 2 β (95% CI)	p	Model 3 β (95% CI)	p
AUCg	-<0.0013 (-<0.001, <0.001)	0.12	**-<0.0015 (-<0.001, -<0.001)**	**0.012**	**-<0.0015 (-<0.001, -<0.001)**	**0.014**

n = Model 1: 3618; Model 2: 3250; Model 3: 3249

Predictor	Model 1 β (95% CI)	p	Model 2 β (95% CI)	p	Model 3 β (95% CI)	p
AUCi (total change)	-<0.0012 (-<0.001, -<0.001)	0.16	-<0.0011 (-<0.001, <0.001)	0.57	-<0.0011 (-<0.001, <0.001)	0.56

n = Model 1: 3618; Model 2: 3250; Model 3: 3249

Predictor	Model 1 β (95% CI)	p	Model 2 β (95% CI)	p	Model 3 β (95% CI)	p
Diurnal slope	-0.14 (-0.33, 0.05)	0.14	-0.09 (-0.27, 0.10)	0.36	-0.07 (-0.26, 0.11)	0.44

n = Model 1: 3618; Model 2: 3250; Model 3: 3249

© 2026 Ng TS et al. *JAMA Network Open*.

Notes: β reflects association with annual rate of cognitive change (lag terms). Significant results ($p < 0.05$) are bolded. Model 1 controlled for demographics (age, sex, education, and race). Model 2 additionally included health factors (chronic conditions, BMI, APOE ε4, antidepressant and glucocorticoid use). Model 3 further added health behaviors (smoking, alcohol use). Significant results ($p < 0.05$) are bolded.

Abbreviations: AUCg (area under the curve, ground); AUCi (area under the curve, increase)

© 2026 Ng TS et al. *JAMA Network Open*.

eTable 4c. Cross-sectional associations — Linear + Quadratic models

Predictor	Model 1<br>β (95% CI)	p	Model 2<br>β (95% CI)	p	Model 3<br>β (95% CI)	p
Coefficient of variation (CV)	**0.06 (0.04–0.08)**	**<0.001**	**0.05 (0.03–0.07)**	**<0.001**	**0.05 (0.03–0.07)**	**<0.001**
Quadratic term (squared)	**-0.003 (-0.004–-0.002)**	**<0.001**	**-0.002 (-0.004–-0.001)**	**0.001**	**-0.002 (-0.004–-0.001)**	**<0.001**

n = Model 1: 3711; Model 2: 3338; Model 3: 3337

Predictor	Model 1<br>β (95% CI)	p	Model 2<br>β (95% CI)	p	Model 3<br>β (95% CI)	p
Mean cortisol	**-0.06 (-0.10–-0.02)**	**0.01**	-0.03 (-0.08–0.01)	0.16	-0.03 (-0.08–0.01)	0.15
Quadratic term (squared)	0.004 (-<0.001–0.01)	0.06	0.002 (-0.002–0.01)	0.42	0.002 (-0.002–0.01)	0.39

n = Model 1: 3895; Model 2: 3496; Model 3: 3495

Predictor	Model 1<br>β (95% CI)	p	Model 2<br>β (95% CI)	p	Model 3<br>β (95% CI)	p
AUCg	**-0.10 (-0.15–-0.04)**	**<0.001**	**-0.06 (-0.11–-0.002)**	**0.04**	**-0.06 (-0.11–-0.004)**	**0.03**
Quadratic term (squared)	**0.01 (0.003–0.02)**	**0.01**	0.01 (-0.001–0.01)	0.10	0.01 (-0.001–0.01)	0.08

n = Model 1: 3618; Model 2: 3250; Model 3: 3249

Predictor	Model 1<br>β (95% CI)	p	Model 2<br>β (95% CI)	p	Model 3<br>β (95% CI)	p

© 2026 Ng TS et al. *JAMA Network Open*.

AUCi	-0.01 (-0.04–0.02)	0.52	-0.001 (-0.03–0.03)	0.96	0.001 (-0.03–0.03)	0.92
Quadratic term (squared)	-0.001 (-0.002–0.001)	0.36	-0.0002 (-0.002–0.002)	0.86	-0.0002 (-0.002–0.002)	0.85

n = Model 1: 3618; Model 2: 3250; Model 3: 3249

Predictor	Model 1 β (95% CI)	p	Model 2 β (95% CI)	p	Model 3 β (95% CI)	p
Diurnal slope	-0.99 (-2.88–0.89)	0.30	-1.00 (-2.95–0.94)	0.31	-0.98 (-2.92–0.96)	0.32
Quadratic term (squared)	2.15 (-10.92–15.21)	0.75	1.07 (-12.35–14.48)	0.88	0.85 (-12.51–14.20)	0.90

n = Model 1: 3618; Model 2: 3250; Model 3: 3249

Notes: Models include both the linear term and its quadratic term simultaneously. Same covariate adjustments as eTable 4a. Model 1 controlled for demographics (age, sex, education, and race). Model 2 additionally included health factors (chronic conditions, BMI, APOE ε4, antidepressant and glucocorticoid use). Model 3 further added health behaviors (smoking, alcohol use). Significant results ($p < 0.05$) are bolded.

Abbreviations: AUCg (area under the curve, ground); AUCi (area under the curve, increase)

© 2026 Ng TS et al. *JAMA Network Open*.

eTable 4d. Longitudinal associations (rate of cognitive decline) — Linear + Quadratic models

Predictor	Model 1 β (95% CI)	p	Model 2 β (95% CI)	p	Model 3 β (95% CI)	p
Coefficient of variation (CV)	**0.01 (0.002–0.01)**	**0.01**	**0.01 (0.003–0.02)**	**0.004**	**0.01 (0.003–0.01)**	**0.004**
Quadratic term (squared)	**-<0.001 (-0.001–-<0.001)**	**0.03**	**-0.001 (-0.001–-<0.001)**	**0.01**	**-0.001 (-0.001–-<0.001)**	**0.01**

n = Model 1: 3711; Model 2: 3338; Model 3: 3337

Predictor	Model 1 β (95% CI)	p	Model 2 β (95% CI)	p	Model 3 β (95% CI)	p
Mean cortisol	-0.01 (-0.03–0.005)	0.18	-0.01 (-0.03–0.002)	0.10	-0.01 (-0.03–0.003)	0.12
Quadratic term (squared)	0.001 (-0.001–0.003)	0.33	0.001 (-0.001–0.002)	0.39	0.001 (-0.001–0.002)	0.44

n = Model 1: 3895; Model 2: 3496; Model 3: 3495

Predictor	Model 1 β (95% CI)	p	Model 2 β (95% CI)	p	Model 3 β (95% CI)	p
AUCg	-0.003 (-0.02–0.01)	0.71	-0.01 (-0.02–0.01)	0.47	-0.01 (-0.02–0.01)	0.51
Quadratic term (squared)	-<0.001 (-0.002–0.002)	0.85	-<0.001 (-0.002–0.002)	0.82	-<0.001 (-0.002–0.002)	0.78

n = Model 1: 3618; Model 2: 3250; Model 3: 3249

Predictor	Model 1 β (95% CI)	p	Model 2 β (95% CI)	p	Model 3 β (95% CI)	p

© 2026 Ng TS et al. *JAMA Network Open*.

AUCi	-0.01 (-0.01–0.004)	0.24	-0.003 (-0.01–0.01)	0.52	-0.003 (-0.01–0.01)	0.52
Quadratic term (squared)	-0.0001 (-0.001–0.001)	0.62	-0.0002 (-0.001–0.001)	0.75	-0.0002 (-0.001–0.001)	0.74

n = Model 1: 3618; Model 2: 3250; Model 3: 3249

Predictor	Model 1 β (95% CI)	p	Model 2 β (95% CI)	p	Model 3 β (95% CI)	p
Diurnal slope	-0.27 (-0.86–0.32)	0.36	-0.20 (-0.79–0.38)	0.49	-0.17 (-0.76–0.41)	0.56
Quadratic term (squared)	-0.91 (-4.79–2.97)	0.65	-0.81 (-4.66–3.03)	0.68	-0.71 (-4.55–3.14)	0.72

n = Model 1: 3618; Model 2: 3250; Model 3: 3249

Notes: Model 1 controlled for demographics (age, sex, education, and race). Model 2 additionally included health factors (chronic conditions, BMI, APOE ε4, antidepressant and glucocorticoid use). Model 3 further added health behaviors (smoking, alcohol use). Significant results ($p < 0.05$) are bolded.

Abbreviations: AUCg (area under the curve, ground); AUCi (area under the curve, increase)

© 2026 Ng TS et al. *JAMA Network Open*.

eTable 5a. Associations of baseline cortisol indices with incident Alzheimer disease — Continuous (Cox models)

Predictor	Model 1<br>OR (95% CI)	Model 2<br>OR (95% CI)	Model 3<br>OR (95% CI)
Coefficient of variation (CV)	0.93 (0.86,1.01)	0.93 (0.86–1.01)	0.93 (0.85–1.01)
Mean cortisol	0.90 (0.59,1.35)	0.86 (0.52–1.43)	0.89 (0.57–1.38)
AUCg	0.96 (0.68,1.34)	0.98 (0.71–1.36)	1.00 (0.72–1.38)
AUCi	1.03 (0.71,1.50)	1.05 (0.74–1.49)	1.05 (0.73–1.53)
Diurnal slope	1.05 (0.78,1.40)	1.04 (0.74–1.46)	1.04 (0.75–1.46)

Notes: Model 1 controlled for demographics (age, sex, education, and race). Model 2 additionally included health factors (chronic conditions, BMI, APOE ε4, antidepressant and glucocorticoid use). Model 3 further added health behaviors (smoking, alcohol use). Significant results ($p < 0.05$) are bolded.

Abbreviations: AUCg (area under the curve, ground); AUCi (area under the curve, increase)

© 2026 Ng TS et al. *JAMA Network Open*.

eTable 5b. Associations of baseline cortisol indices with incident Alzheimer disease — Quintiles (Ref = Q1)

Predictor & Quintile	Model 1<br>OR (95% CI)	Model 2<br>OR (95% CI)	Model 3<br>OR (95% CI)
Coefficient of variation (CV)			
Q2 vs Q1	2.52 (0.93,6.83)	2.56 (0.87–7.52)	2.40(0.79–7.24)
Q3 vs Q1	1.61 (0.27,9.47)	1.52 (0.25–9.22)	1.41 (0.22–9.18)
Q4 vs Q1	1.39 (0.54,3.59)	1.42 (0.52–3.85)	1.55 (0.54–4.45)
Q5 vs Q1	0.57 (0.22,1.51)	0.53 (0.19–1.49)	0.48 (0.16–1.44)
Mean cortisol			
Q2 vs Q1	0.90 (0.28,2.82)	0.94 (0.25–3.49)	0.92 (0.254–3.59)
Q3 vs Q1	2.29 (0.59,8.96)	2.75 (0.64–11.91)	2.98 (0.68–13.06)
Q4 vs Q1	2.24 (0.58,8.69)	2.60 (0.65–10.36)	2.45 (0.61–9.94)
Q5 vs Q1	1.46 (0.43,4.90)	1.49 (0.40–5.54)	1.67 (0.46–6.10)
AUCg			
Q2 vs Q1	0.74 (0.20,2.70)	0.64 (0.15–2.69)	0.62 (0.13–2.94)
Q3 vs Q1	2.30 (0.52,10.20)	2.44 (0.54–11.16)	2.42 (0.50–11.67)
Q4 vs Q1	1.52 (0.44,5.23)	1.52 (0.41–5.62)	1.47 (0.39–5.53)
Q5 vs Q1	1.44 (0.40,5.14)	1.50 (0.40–5.54)	1.61 (0.40–5.49)
AUCi			
Q2 vs Q1	2.17 (0.64,7.36)	2.21 (0.63–7.74)	2.03 (0.61–6.80)
Q3 vs Q1	2.49 (0.49,12.55)	2.41 (0.49–11.91)	2.15 (0.46–10.12)
Q4 vs Q1	1.85 (0.55,6.28)	2.01 (0.55–7.30)	1.89 (0.52–6.92)
Q5 vs Q1	1.73 (0.55,5.39)	1.71 (0.52–5.70)	1.67 (0.49–5.73)
Diurnal slope			
Q2 vs Q1	2.45 (0.65,9.29)	2.53(0.59–10.76)	2.72 (0.63–11.83)

© 2026 Ng TS et al. *JAMA Network Open*.

Q3 vs Q1	1.34 (0.36,5.08)	1.24 (0.29–5.7)	1.32 (0.27–6.34)
Q4 vs Q1	2.81(0.77,10.18)	2.94 (0.69–12.45)	2.88 (0.69–12.09)
Q5 vs Q1	1.27 (0.40,4.00)	1.25 (0.39–4.01)	1.30 (0.36–4.70)

Notes: Model 1 controlled for demographics (age, sex, education, and race). Model 2 additionally included health factors (chronic conditions, BMI, APOE ε4, antidepressant and glucocorticoid use). Model 3 further added health behaviors (smoking, alcohol use). Significant results ($p < 0.05$) are bolded.

Abbreviations: AUCg (area under the curve, ground); AUCi (area under the curve, increase)

© 2026 Ng TS et al. *JAMA Network Open*.

eTable 6a. Global Cognition and Salivary Cortisol Indices: Race × Cortisol Interaction Models

i. Cross-sectional Associations

CV — Concurrent (Cross-sectional)

Predictor	Model 1 β (95% CI)	p	Model 2 β (95% CI)	p	Model 3 β (95% CI)	p
**Q2**	**0.14 (0.03 to 0.26)**	**0.015**	**0.17 (0.05 to 0.28)**	**0.004**	**0.17 (0.06 to 0.28)**	**0.003**
**Q3**	**0.17 (0.06 to 0.29)**	**0.003**	**0.17 (0.06 to 0.28)**	**0.002**	**0.17 (0.06 to 0.28)**	**0.003**
**Q4**	**0.17 (0.06 to 0.28)**	**0.002**	**0.17 (0.07 to 0.28)**	**0.001**	**0.17 (0.07 to 0.27)**	**0.001**
**Q5**	**0.16 (0.05 to 0.27)**	**0.003**	**0.17 (0.07 to 0.28)**	**0.001**	**0.17 (0.06 to 0.27)**	**0.002**
Black × CV Q2	-0.06 (-0.20 to 0.07)	0.38	-0.11 (-0.25 to 0.02)	0.09	-0.12 (-0.25 to 0.01)	0.08
Black × CV Q3	0.02 (-0.11 to 0.16)	0.71	0.002 (-0.131 to 0.128)	0.98	0.002 (-0.131 to 0.127)	0.97
Black × CV Q4	-0.02 (-0.15 to 0.11)	0.81	-0.08 (-0.20 to 0.05)	0.24	-0.07 (-0.20 to 0.05)	0.25
Black × CV Q5	0.01 (-0.12 to 0.14)	0.88	-0.04 (-0.16 to 0.09)	0.56	-0.03 (-0.16 to 0.09)	0.63

Mean Cortisol — Concurrent (Cross-sectional)

Predictor	Model 1 β (95% CI)	p	Model 2 β (95% CI)	p	Model 3 β (95% CI)	p
Q2	0.05 (-0.05 to 0.15)	0.33	0.04 (-0.06 to 0.13)	0.45	0.03 (-0.07 to 0.13)	0.53
Q3	0.02 (-0.08 to 0.12)	0.66	0.02 (-0.08 to 0.12)	0.68	0.01 (-0.08 to 0.11)	0.82
Q4	-0.02 (-0.12 to 0.07)	0.62	-0.03 (-0.12 to 0.07)	0.57	-0.03 (-0.12 to 0.06)	0.53

© 2026 Ng TS et al. *JAMA Network Open*.

Q5	-0.05 (-0.15 to 0.05)	0.29	-0.05 (-0.15 to 0.05)	0.32	-0.05 (-0.15 to 0.04)	0.28
Black × Mean Q2	-0.02 (-0.15 to 0.10)	0.70	-0.01 (-0.13 to 0.11)	0.81	-0.01 (-0.13 to 0.11)	0.86
Black × Mean Q3	-0.01 (-0.13 to 0.11)	0.85	-0.02 (-0.14 to 0.10)	0.76	-0.01 (-0.13 to 0.11)	0.83
Black × Mean Q4	0.03 (-0.09 to 0.15)	0.67	0.03 (-0.09 to 0.15)	0.65	0.03 (-0.09 to 0.15)	0.65
Black × Mean Q5	-0.09 (-0.21 to 0.03)	0.14	-0.05 (-0.17 to 0.07)	0.42	-0.05 (-0.17 to 0.07)	0.44

AUCg — Concurrent (Cross-sectional)

Predictor	Model 1 β (95% CI)	p	Model 2 β (95% CI)	p	Model 3 β (95% CI)	p
Q2	-0.004 (-0.109 to 0.101)	0.94	-0.03 (-0.13 to 0.08)	0.62	-0.03 (-0.13 to 0.07)	0.58
Q3	0.05 (-0.05 to 0.15)	0.37	0.05 (-0.05 to 0.15)	0.35	0.04 (-0.06 to 0.14)	0.40
Q4	-0.08 (-0.18 to 0.02)	0.12	-0.08 (-0.18 to 0.02)	0.10	-0.08 (-0.18 to 0.01)	0.10
Q5	-0.05 (-0.15 to 0.05)	0.33	-0.05 (-0.15 to 0.05)	0.32	-0.05 (-0.15 to 0.05)	0.30
Black × AUCg Q2	0.04 (-0.09 to 0.16)	0.55	0.06 (-0.06 to 0.19)	0.33	0.06 (-0.06 to 0.19)	0.31
Black × AUCg Q3	-0.01 (-0.14 to 0.11)	0.84	-0.01 (-0.14 to 0.11)	0.82	-0.01 (-0.13 to 0.11)	0.86
Black × AUCg Q4	0.09 (-0.04 to 0.21)	0.16	0.10 (-0.02 to 0.22)	0.10	0.10 (-0.02 to 0.22)	0.11
Black × AUCg Q5	-0.09 (-0.21 to 0.04)	0.18	-0.04 (-0.16 to 0.08)	0.55	-0.04 (-0.16 to 0.09)	0.56

Slope — Concurrent (Cross-sectional)

Predictor	Model 1 β (95% CI)	p	Model 2 β (95% CI)	p	Model 3 β (95% CI)	p

© 2026 Ng TS et al. *JAMA Network Open*.

Q2	0.02 (-0.07 to 0.11)	0.69	0.01 (-0.08 to 0.09)	0.88	0.01 (-0.07 to 0.10)	0.74
Q3	-0.04 (-0.14 to 0.05)	0.39	-0.04 (-0.13 to 0.05)	0.38	-0.03 (-0.12 to 0.06)	0.49
Q4	-0.02 (-0.12 to 0.08)	0.67	-0.04 (-0.14 to 0.05)	0.38	-0.03 (-0.13 to 0.07)	0.55
Q5	-0.03 (-0.13 to 0.07)	0.56	-0.04 (-0.14 to 0.06)	0.42	-0.03 (-0.13 to 0.07)	0.56
Black × Slope Q2	-0.05 (-0.16 to 0.07)	0.45	-0.03 (-0.15 to 0.08)	0.58	-0.04 (-0.15 to 0.08)	0.51
Black × Slope Q3	0.01 (-0.11 to 0.13)	0.86	0.05 (-0.06 to 0.17)	0.37	0.04 (-0.07 to 0.16)	0.46
Black × Slope Q4	-0.09 (-0.21 to 0.03)	0.15	-0.04 (-0.16 to 0.08)	0.50	-0.06 (-0.18 to 0.06)	0.36
Black × Slope Q5	**-0.13 (-0.26 to 0.004)**	**0.043**	-0.09 (-0.22 to 0.03)	0.15	-0.10 (-0.23 to 0.02)	0.11

AUCi — Concurrent (Cross-sectional)

Predictor	Model 1 β (95% CI)	p	Model 2 β (95% CI)	p	Model 3 β (95% CI)	p
Q2	0.01 (-0.07 to 0.10)	0.79	0.001 (-0.083 to 0.085)	0.98	0.000 (-0.084 to 0.083)	1.00
Q3	-0.01 (-0.11 to 0.08)	0.80	0.001 (-0.090 to 0.091)	0.99	0.01 (-0.08 to 0.10)	0.88
Q4	-0.01 (-0.11 to 0.09)	0.85	-0.02 (-0.12 to 0.08)	0.65	-0.01 (-0.11 to 0.08)	0.78
Q5	-0.09 (-0.19 to 0.01)	0.10	-0.09 (-0.19 to 0.01)	0.07	-0.08 (-0.18 to 0.02)	0.10
Black × AUCi Q2	0.02 (-0.10 to 0.13)	0.80	-0.005 (-0.120 to 0.111)	0.94	-0.001 (-0.115 to 0.114)	0.99
Black × AUCi Q3	0.03 (-0.10 to 0.15)	0.69	0.01 (-0.11 to 0.12)	0.92	0.002 (-0.116 to 0.120)	0.97
Black × AUCi Q4	-0.05 (-0.18 to 0.07)	0.43	-0.03 (-0.15 to 0.10)	0.68	-0.03 (-0.16 to 0.09)	0.59

© 2026 Ng TS et al. *JAMA Network Open*.

Black × AUCi Q5	-0.08 (-0.21 to 0.05)	0.21	-0.06 (-0.18 to 0.07)	0.37	-0.06 (-0.19 to 0.06)	0.32

Notes: *Model 1 controlled for demographics (age, sex, education, and race). Model 2 additionally included health factors (chronic conditions, BMI, APOE ε4, antidepressant and glucocorticoid use). Model 3 further added health behaviors (smoking, alcohol use). Significant results ($p < 0.05$) are bolded.*

Abbreviations: AUCg (area under the curve, ground); AUCi (area under the curve, increase)

© 2026 Ng TS et al. *JAMA Network Open*.

ii. Longitudinal association

CV — Lagged (Longitudinal)

Predictor	Model 1 β (95% CI)	p	Model 2 β (95% CI)	p	Model 3 β (95% CI)	p
Q2	0.02 (-0.02 to 0.06)	0.30	0.02 (-0.02 to 0.06)	0.32	0.02 (-0.02 to 0.06)	0.33
**Q3**	**0.04 (0.01 to 0.08)**	**0.019**	**0.04 (0.01 to 0.08)**	**0.016**	**0.04 (0.01 to 0.08)**	**0.020**
**Q4**	**0.04 (0.01 to 0.08)**	**0.018**	**0.04 (0.01 to 0.08)**	**0.022**	**0.04 (0.01 to 0.08)**	**0.024**
Q5	0.03 (0.005 to 0.06)	0.09	0.03 (-0.01 to 0.06)	0.14	0.02 (-0.01 to 0.06)	0.15
Black × CV Q2	0.004 (-0.049 to 0.040)	0.85	0.000 (-0.043 to 0.044)	0.99	0.001 (-0.045 to 0.043)	0.97
Black × CV Q3	-0.04 (-0.08 to 0.01)	0.10	-0.04 (-0.08 to 0.01)	0.09	-0.03 (-0.08 to 0.01)	0.11
Black × CV Q4	-0.02 (-0.07 to 0.02)	0.26	-0.02 (-0.06 to 0.02)	0.39	-0.02 (-0.06 to 0.02)	0.42
Black × CV Q5	-0.01 (-0.05 to 0.03)	0.61	-0.01 (-0.05 to 0.03)	0.51	-0.01 (-0.05 to 0.03)	0.60

Mean Cortisol — Lagged (Longitudinal)

Predictor	Model 1 β (95% CI)	p	Model 2 β (95% CI)	p	Model 3 β (95% CI)	p
Q2	0.002 (-0.026 to 0.030)	0.88	0.003 (-0.024 to 0.030)	0.83	0.001 (-0.026 to 0.028)	0.94
Q3	-0.01 (-0.03 to 0.02)	0.72	-0.003 (-0.030 to 0.023)	0.81	-0.004 (-0.031 to 0.022)	0.74
Q4	-0.003 (-0.032 to 0.026)	0.82	-0.004 (-0.032 to 0.024)	0.78	-0.01 (-0.03 to 0.02)	0.67
**Q5**	**-0.02 (-0.05 to 0.01)**	**0.12**	**-0.03 (-0.06 to 0.00)**	**0.052**	**-0.03 (-0.06 to -0.00)**	**0.04**

© 2026 Ng TS et al. *JAMA Network Open*.

Black × Mean Q2	-0.01 (-0.04 to 0.03)	0.67	-0.005 (-0.037 to 0.027)	0.77	-0.003 (-0.035 to 0.029)	0.85
Black × Mean Q3	0.001 (-0.032 to 0.035)	0.95	0.003 (-0.029 to 0.036)	0.84	0.01 (-0.03 to 0.04)	0.67
Black × Mean Q4	-0.01 (-0.04 to 0.03)	0.71	0.001 (-0.033 to 0.035)	0.96	0.003 (-0.032 to 0.037)	0.87
Black × Mean Q5	0.01 (-0.02 to 0.05)	0.47	0.01 (-0.02 to 0.05)	0.52	0.02 (-0.02 to 0.05)	0.40

AUCg — Lagged (Longitudinal)

Predictor	Model 1 β (95% CI)	p	Model 2 β (95% CI)	p	Model 3 β (95% CI)	p
Q2	0.004 (-0.023 to 0.031)	0.78	0.003 (-0.023 to 0.030)	0.81	0.003 (-0.023 to 0.030)	0.80
Q3	0.001 (-0.028 to 0.030)	0.94	0.002 (-0.026 to 0.030)	0.89	0.001 (-0.027 to 0.029)	0.95
Q4	0.01 (-0.02 to 0.04)	0.58	0.01 (-0.02 to 0.04)	0.65	0.005 (-0.024 to 0.033)	0.74
Q5	-0.02 (-0.05 to 0.01)	0.28	-0.02 (-0.05 to 0.01)	0.15	-0.02 (-0.05 to 0.01)	0.12
Black × AUCg Q2	-0.02 (-0.05 to 0.02)	0.35	-0.01 (-0.05 to 0.02)	0.38	-0.01 (-0.05 to 0.02)	0.45
Black × AUCg Q3	-0.01 (-0.04 to 0.03)	0.75	0.003 (-0.037 to 0.030)	0.85	0.002 (-0.035 to 0.032)	0.92
Black × AUCg Q4	-0.02 (-0.06 to 0.02)	0.28	-0.01 (-0.05 to 0.02)	0.53	-0.01 (-0.04 to 0.03)	0.70
Black × AUCg Q5	0.01 (-0.03 to 0.04)	0.71	0.01 (-0.03 to 0.04)	0.72	0.01 (-0.03 to 0.04)	0.63

Slope — Lagged (Longitudinal)

Predictor	Model 1 β (95% CI)	p	Model 2 β (95% CI)	p	Model 3 β (95% CI)	p
Q2	0.01 (-0.02 to 0.03)	0.62	0.01 (-0.02 to 0.03)	0.46	0.01 (-0.02 to 0.03)	0.49

© 2026 Ng TS et al. *JAMA Network Open*.

Q3	0.000 (-0.028 to 0.029)	0.98	0.002 (-0.026 to 0.029)	0.90	0.001 (-0.027 to 0.029)	0.93
Q4	-0.01 (-0.04 to 0.03)	0.74	-0.004 (-0.035 to 0.026)	0.78	-0.002 (-0.033 to 0.028)	0.88
Q5	-0.02 (-0.05 to 0.01)	0.28	-0.01 (-0.04 to 0.02)	0.52	-0.01 (-0.04 to 0.02)	0.52
Black × Slope Q2	-0.01 (-0.05 to 0.02)	0.47	-0.01 (-0.04 to 0.02)	0.65	-0.01 (-0.04 to 0.03)	0.68
Black × Slope Q3	-0.01 (-0.05 to 0.02)	0.42	-0.01 (-0.05 to 0.02)	0.50	-0.01 (-0.05 to 0.02)	0.50
Black × Slope Q4	-0.004 (-0.041 to 0.033)	0.83	-0.001 (-0.037 to 0.036)	0.97	-0.003 (-0.039 to 0.034)	0.89
Black × Slope Q5	-0.002 (-0.038 to 0.034)	0.92	-0.001 (-0.037 to 0.034)	0.94	0.001 (-0.034 to 0.036)	0.96

AUCi — Lagged (Longitudinal)

Predictor	Model 1 β (95% CI)	p	Model 2 β (95% CI)	p	Model 3 β (95% CI)	p
Q2	-0.01 (-0.03 to 0.02)	0.63	0.001 (-0.025 to 0.026)	0.96	0.000 (-0.026 to 0.025)	0.98
Q3	-0.01 (-0.04 to 0.02)	0.57	-0.003 (-0.030 to 0.025)	0.85	-0.003 (-0.031 to 0.024)	0.81
Q4	-0.02 (-0.05 to 0.01)	0.20	-0.02 (-0.05 to 0.01)	0.27	-0.02 (-0.05 to 0.01)	0.30
Q5	0.001 (-0.029 to 0.032)	0.93	0.01 (-0.02 to 0.04)	0.68	0.01 (-0.02 to 0.03)	0.72
Black × AUCi Q2	0.03 (-0.01 to 0.06)	0.10	0.03 (-0.01 to 0.06)	0.12	0.03 (-0.01 to 0.06)	0.11
Black × AUCi Q3	0.01 (-0.03 to 0.04)	0.73	0.01 (-0.02 to 0.05)	0.53	0.01 (-0.02 to 0.05)	0.53
Black × AUCi Q4	0.02 (-0.01 to 0.06)	0.23	0.03 (-0.01 to 0.06)	0.15	0.03 (-0.01 to 0.06)	0.18
Black × AUCi Q5	-0.003 (-0.040 to 0.035)	0.89	-0.001 (-0.037 to 0.035)	0.96	0.002 (-0.034 to 0.038)	0.91

© 2026 Ng TS et al. *JAMA Network Open*.

Notes: *Model 1 controlled for demographics (age, sex, education, and race). Model 2 additionally included health factors (chronic conditions, BMI, APOE ε4, antidepressant and glucocorticoid use). Model 3 further added health behaviors (smoking, alcohol use). Significant results (p < 0.05) are bolded.*

Abbreviations: AUCg (area under the curve, ground); AUCi (area under the curve, increase)

eTable 6b — Race-Stratified Models: Global Cognition and Salivary Cortisol Indices

Black Older Adults — Concurrent (Cross-sectional)

CV

Predictor	Model 1<br>β (95% CI)	p	Model 2<br>β (95% CI)	p	Model 3<br>β (95% CI)	p
Q2	**0.08 (0.01 to 0.15)**	**0.027**	0.05 (-0.02 to 0.12)	0.18	0.05 (-0.02 to 0.12)	0.17
**Q3**	**0.19 (0.12 to 0.26)**	**< 0.001**	**0.16 (0.09 to 0.23)**	**< 0.001**	**0.16 (0.09 to 0.23)**	**< 0.001**
**Q4**	**0.15 (0.07 to 0.22)**	**< 0.001**	**0.09 (0.02 to 0.17)**	**0.017**	**0.09 (0.01 to 0.16)**	**0.020**
**Q5**	**0.16 (0.08 to 0.23)**	**< 0.001**	**0.13 (0.05 to 0.20)**	**0.001**	**0.12 (0.05 to 0.20)**	**0.002**

Mean

Predictor	Model 1<br>β (95% CI)	p	Model 2<br>β (95% CI)	p	Model 3<br>β (95% CI)	p
Q2	0.03 (-0.05 to 0.10)	0.49	0.02 (-0.05 to 0.09)	0.57	0.02 (-0.05 to 0.09)	0.62
Q3	0.01 (-0.06 to 0.09)	0.73	0.003 (-0.070 to 0.076)	0.93	0.001 (-0.072 to 0.073)	0.99
Q4	0.001 (-0.074 to 0.077)	0.97	0.002 (-0.074 to 0.077)	0.97	-0.0003 (-0.076 to 0.075)	0.99
**Q5**	**-0.14 (-0.22 to -0.06)**	**< 0.001**	**-0.10 (-0.17 to -0.02)**	**0.013**	**-0.09 (-0.17 to -0.02)**	**0.013**

AUCg

Predictor	Model 1<br>β (95% CI)	p	Model 2<br>β (95% CI)	p	Model 3<br>β (95% CI)	p
Q2	0.03 (-0.04 to 0.11)	0.36	0.04 (-0.04 to 0.11)	0.34	0.03 (-0.04 to 0.11)	0.37

© 2026 Ng TS et al. *JAMA Network Open*.

Q3	0.03 (-0.04 to 0.11)	0.38	0.03 (-0.04 to 0.11)	0.39	0.03 (-0.04 to 0.11)	0.41
Q4	0.01 (-0.07 to 0.09)	0.83	0.02 (-0.05 to 0.10)	0.57	0.02 (-0.06 to 0.10)	0.58
**Q5**	**-0.13 (-0.21 to -0.05)**	**< 0.001**	**-0.08 (-0.16 to -0.01)**	**0.035**	**-0.08 (-0.16 to -0.01)**	**0.04**

Slope

Predictor	Model 1<br>β (95% CI)	p	Model 2<br>β (95% CI)	p	Model 3<br>β (95% CI)	p
Q2	-0.02 (-0.11 to 0.06)	0.59	-0.02 (-0.10 to 0.06)	0.58	-0.02 (-0.10 to 0.06)	0.61
Q3	-0.02 (-0.10 to 0.06)	0.65	0.02 (-0.06 to 0.10)	0.57	0.02 (-0.06 to 0.10)	0.55
**Q4**	**-0.10 (-0.18 to -0.02)**	**0.013**	**-0.08 (-0.16 to 0.001)**	**0.053**	**-0.08 (-0.16 to 0.002)**	**0.046**
**Q5**	**-0.15 (-0.23 to -0.07)**	**< 0.001**	**-0.12 (-0.20 to -0.04)**	**0.003**	**-0.12 (-0.20 to -0.04)**	**0.003**

AUCi

Predictor	Model 1<br>β (95% CI)	p	Model 2<br>β (95% CI)	p	Model 3<br>β (95% CI)	p
Q2	0.000 (-0.060 to 0.061)	0.99	0.002 (-0.084 to 0.080)	0.96	0.000 (-0.081 to 0.082)	0.99
Q3	-0.04 (-0.10 to 0.02)	0.19	0.01 (-0.07 to 0.09)	0.83	0.01 (-0.07 to 0.09)	0.77
Q4	-0.11 (-0.17 to -0.05)	< 0.001	-0.04 (-0.12 to 0.03)	0.26	-0.04 (-0.12 to 0.03)	0.27
**Q5**	**-0.21 (-0.27 to -0.15)**	**< 0.001**	**-0.13 (-0.21 to -0.05)**	**0.001**	**-0.13 (-0.21 to -0.05)**	**0.001**

© 2026 Ng TS et al. *JAMA Network Open*.

Black Older Adults — Lagged (Longitudinal)

CV

Predictor	Model 1 β (95% CI)	p	Model 2 β (95% CI)	p	Model 3 β (95% CI)	p
Q2	0.02 (-0.01 to 0.04)	0.15	0.02 (0.003 to 0.04)	0.09	0.02 (0.004 to 0.04)	0.11
Q3	0.01 (-0.01 to 0.03)	0.50	0.01 (-0.01 to 0.03)	0.55	0.01 (-0.01 to 0.03)	0.54
Q4	0.02 (0.002 to 0.04)	0.07	**0.02 (0.002 to 0.04)**	**0.032**	**0.02 (0.002 to 0.04)**	**0.03**
Q5	0.02 (0.002 to 0.04)	0.07	0.01 (-0.01 to 0.03)	0.34	0.01 (-0.01 to 0.03)	0.26

Mean

Predictor	Model 1 β (95% CI)	p	Model 2 β (95% CI)	p	Model 3 β (95% CI)	p
Q2	-0.01 (-0.02 to 0.01)	0.60	-0.002 (-0.020 to 0.016)	0.85	-0.002 (-0.020 to 0.016)	0.83
Q3	-0.004 (-0.024 to 0.016)	0.70	-0.000 (-0.019 to 0.019)	1.00	0.002 (-0.017 to 0.021)	0.82
Q4	-0.01 (-0.03 to 0.01)	0.38	-0.003 (-0.024 to 0.017)	0.76	-0.003 (-0.024 to 0.017)	0.74
Q5	-0.01 (-0.03 to 0.01)	0.40	-0.02 (-0.04 to 0.004)	0.12	-0.01 (-0.04 to 0.01)	0.16

AUCg

Predictor	Model 1 β (95% CI)	p	Model 2 β (95% CI)	p	Model 3 β (95% CI)	p
Q2	-0.003 (-0.022 to 0.016)	0.75	0.001 (-0.018 to 0.019)	0.94	0.001 (-0.018 to 0.019)	0.95
Q3	-0.004 (-0.024 to 0.016)	0.68	-0.001 (-0.020 to 0.019)	0.96	-0.000 (-0.020 to 0.019)	0.97
Q4	-0.01 (-0.03 to 0.01)	0.34	-0.005 (-0.026 to 0.017)	0.66	-0.003 (-0.024 to 0.019)	0.82
Q5	-0.01 (-0.03 to 0.01)	0.41	-0.01 (-0.04 to 0.01)	0.17	-0.01 (-0.04 to 0.01)	0.18

© 2026 Ng TS et al. *JAMA Network Open*.

Slope

Predictor	Model 1<br>β (95% CI)	p	Model 2<br>β (95% CI)	p	Model 3<br>β (95% CI)	p
Q2	-0.01 (-0.03 to 0.01)	0.53	0.003 (-0.018 to 0.023)	0.79	0.002 (-0.018 to 0.023)	0.84
Q3	-0.02 (-0.04 to 0.01)	0.15	-0.01 (-0.03 to 0.01)	0.33	-0.01 (-0.03 to 0.01)	0.28
Q4	-0.01 (-0.03 to 0.01)	0.34	-0.005 (-0.025 to 0.016)	0.66	-0.004 (-0.025 to 0.016)	0.67
Q5	-0.02 (-0.04 to 0.003)	0.09	-0.01 (-0.03 to 0.01)	0.43	-0.01 (-0.03 to 0.01)	0.56

AUCi

Predictor	Model 1<br>β (95% CI)	p	Model 2<br>β (95% CI)	p	Model 3<br>β (95% CI)	p
Q2	0.01 (-0.01 to 0.03)	0.23	**0.03 (0.004 to 0.05)**	**0.02**	**0.03 (0.004 to 0.05)**	**0.02**
Q3	-0.01 (-0.02 to 0.01)	0.44	0.01 (-0.01 to 0.03)	0.45	0.01 (-0.01 to 0.03)	0.49
Q4	-0.01 (-0.02 to 0.01)	0.41	0.01 (-0.01 to 0.03)	0.32	0.01 (-0.01 to 0.03)	0.35
Q5	-0.01 (-0.02 to 0.01)	0.57	0.01 (-0.02 to 0.03)	0.60	0.01 (-0.01 to 0.03)	0.47

Notes: *Model 1 controlled for demographics (age, sex, education, and race). Model 2 additionally included health factors (chronic conditions, BMI, APOE ε4, antidepressant and glucocorticoid use). Model 3 further added health behaviors (smoking, alcohol use). Significant results ($p < 0.05$) are bolded.*

Abbreviations: AUCg (area under the curve, ground); AUCi (area under the curve, increase)

© 2026 Ng TS et al. *JAMA Network Open*.

White Older Adults — Concurrent (Cross-sectional)

CV

Variable	β (95% CI) all1a	p	β (95% CI) all1b	p	β (95% CI) all1c	p
**Q2**	**0.14 (0.04 to 0.24)**	**0.008**	**0.16 (0.06 to 0.27)**	**0.003**	**0.17 (0.06 to 0.27)**	**0.002**
**Q3**	**0.17 (0.07 to 0.27)**	**0.001**	**0.17 (0.07 to 0.27)**	**0.001**	**0.16 (0.06 to 0.26)**	**0.002**
**Q4**	**0.17 (0.08 to 0.27)**	**< 0.001**	**0.18 (0.08 to 0.27)**	**< 0.001**	**0.17 (0.07 to 0.26)**	**< 0.001**
**Q5**	**0.18 (0.08 to 0.27)**	**< 0.001**	**0.18 (0.09 to 0.28)**	**< 0.001**	**0.17 (0.07 to 0.26)**	**< 0.001**

Mean

Variable	β (95% CI) all1a	p	β (95% CI) all1b	p	β (95% CI) all1c	p
Q2	0.06 (-0.03 to 0.15)	0.18	0.05 (-0.04 to 0.14)	0.31	0.04 (-0.05 to 0.13)	0.36
Q3	0.03 (-0.06 to 0.12)	0.46	0.03 (-0.06 to 0.12)	0.53	0.02 (-0.07 to 0.11)	0.71
Q4	-0.02 (-0.11 to 0.07)	0.67	-0.02 (-0.11 to 0.06)	0.59	-0.03 (-0.11 to 0.06)	0.56
Q5	-0.05 (-0.14 to 0.04)	0.28	-0.04 (-0.13 to 0.05)	0.34	-0.05 (-0.14 to 0.04)	0.27

AUCg

Variable	β (95% CI) all1a	p	β (95% CI) all1b	p	β (95% CI) all1c	p
Q2	-0.002 (-0.095 to 0.091)	0.96	-0.02 (-0.12 to 0.07)	0.62	-0.02 (-0.12 to 0.07)	0.60
Q3	0.05 (-0.04 to 0.14)	0.31	0.05 (-0.04 to 0.14)	0.30	0.04 (-0.05 to 0.13)	0.34
Q4	-0.07 (-0.16 to 0.01)	0.10	-0.08 (-0.17 to 0.01)	0.08	-0.08 (-0.16 to 0.01)	0.09
Q5	-0.06 (-0.15 to 0.04)	0.23	-0.05 (-0.14 to 0.04)	0.28	-0.05 (-0.14 to 0.04)	0.26

© 2026 Ng TS et al. *JAMA Network Open*.

Slope

Variable	β (95% CI) all1a	p	β (95% CI) all1b	p	β (95% CI) all1c	p
Q2	0.003 (-0.075 to 0.082)	0.93	0.000 (-0.079 to 0.078)	1.00	0.01 (-0.07 to 0.08)	0.85
Q3	-0.07 (-0.15 to 0.02)	0.11	-0.06 (-0.14 to 0.03)	0.17	-0.05 (-0.13 to 0.03)	0.25
Q4	-0.04 (-0.13 to 0.05)	0.36	-0.05 (-0.14 to 0.04)	0.24	-0.04 (-0.12 to 0.05)	0.42
Q5	-0.04 (-0.14 to 0.05)	0.34	-0.05 (-0.14 to 0.04)	0.30	-0.03 (-0.12 to 0.06)	0.50

AUCi

Variable	β (95% CI) all1a	p	β (95% CI) all1b	p	β (95% CI) all1c	p
Q2	0.000 (-0.060 to 0.061)	0.99	0.001 (-0.077 to 0.078)	0.99	-0.001 (-0.077 to 0.075)	0.98
Q3	-0.04 (-0.10 to 0.02)	0.19	-0.01 (-0.09 to 0.08)	0.86	0.004 (-0.079 to 0.086)	0.93
Q4	-0.11 (-0.17 to -0.05)	< 0.001	-0.03 (-0.12 to 0.06)	0.51	-0.02 (-0.11 to 0.07)	0.66
Q5	-0.21 (-0.27 to -0.15)	< 0.001	-0.09 (-0.18 to 0.001)	0.054	-0.07 (-0.16 to 0.01)	0.10

© 2026 Ng TS et al. *JAMA Network Open*.

White Older Adults — Lagged (Longitudinal)

CV

Variable	β (95% CI) all1a	p	β (95% CI) all1b	p	β (95% CI) all1c	p
Q2	0.03 (-0.01 to 0.06)	0.17	0.03 (-0.01 to 0.07)	0.12	0.03 (-0.01 to 0.07)	0.13
**Q3**	**0.05 (0.02 to 0.08)**	**0.005**	**0.05 (0.02 to 0.09)**	**0.003**	**0.05 (0.02 to 0.08)**	**0.005**
**Q4**	**0.05 (0.01 to 0.08)**	**0.005**	**0.05 (0.01 to 0.08)**	**0.005**	**0.05 (0.01 to 0.08)**	**0.005**
Q5	0.03 (0.000 to 0.06)	0.05	0.03 (0.002 to 0.06)	0.06	0.03 (0.003 to 0.06)	0.08

Mean

Variable	β (95% CI) all1a	p	β (95% CI) all1b	p	β (95% CI) all1c	p
Q2	0.002 (-0.023 to 0.027)	0.87	0.000 (-0.026 to 0.025)	0.99	-0.004 (-0.029 to 0.022)	0.79
Q3	-0.004 (-0.029 to 0.021)	0.77	-0.01 (-0.03 to 0.02)	0.70	-0.01 (-0.03 to 0.02)	0.58
Q4	-0.005 (-0.031 to 0.022)	0.73	-0.01 (-0.03 to 0.02)	0.62	-0.01 (-0.04 to 0.02)	0.44
**Q5**	**-0.03 (-0.05 to 0.001)**	**0.056**	**-0.03 (-0.06 to 0.005)**	**0.023**	**-0.04 (-0.06 to -0.01)**	**0.012**

AUCg

Variable	β (95% CI) all1a	p	β (95% CI) all1b	p	β (95% CI) all1c	p
Q2	0.01 (-0.01 to 0.04)	0.30	0.01 (-0.01 to 0.04)	0.37	0.01 (-0.02 to 0.03)	0.55
Q3	0.004 (-0.022 to 0.030)	0.75	0.002 (-0.025 to 0.028)	0.90	-0.001 (-0.027 to 0.026)	0.95
Q4	0.01 (-0.02 to 0.03)	0.64	0.003 (-0.024 to 0.030)	0.83	-0.001 (-0.028 to 0.027)	0.97
Q5	-0.02 (-0.05 to 0.01)	0.18	-0.02 (-0.05 to 0.005)	0.11	-0.03 (-0.05 to 0.001)	0.06

© 2026 Ng TS et al. *JAMA Network Open*.

Slope

Variable	β (95% CI) all1a	p	β (95% CI) all1b	p	β (95% CI) all1c	p
Q2	0.01 (-0.02 to 0.03)	0.50	0.01 (-0.01 to 0.04)	0.40	0.01 (-0.01 to 0.03)	0.43
Q3	0.003 (-0.025 to 0.030)	0.86	0.003 (-0.024 to 0.030)	0.84	0.003 (-0.025 to 0.030)	0.86
Q4	-0.004 (-0.034 to 0.025)	0.77	-0.01 (-0.04 to 0.02)	0.73	-0.002 (-0.032 to 0.028)	0.89
Q5	-0.01 (-0.04 to 0.01)	0.30	-0.01 (-0.04 to 0.02)	0.52	-0.01 (-0.04 to 0.02)	0.57

AUCi

Variable	β (95% CI) all1a	p	β (95% CI) all1b	p	β (95% CI) all1c	p
Q2	0.01 (-0.01 to 0.03)	0.23	-0.002 (-0.027 to 0.023)	0.87	-0.003 (-0.028 to 0.022)	0.82
Q3	-0.01 (-0.02 to 0.01)	0.44	-0.003 (-0.030 to 0.023)	0.81	-0.004 (-0.031 to 0.022)	0.75
Q4	-0.01 (-0.02 to 0.01)	0.41	-0.01 (-0.04 to 0.02)	0.42	-0.01 (-0.04 to 0.02)	0.50
Q5	-0.01 (-0.02 to 0.01)	0.57	0.002 (-0.027 to 0.030)	0.92	0.002 (-0.027 to 0.030)	0.91

Notes: *Model 1 controlled for demographics (age, sex, education, and race). Model 2 additionally included health factors (chronic conditions, BMI, APOE ε4, antidepressant and glucocorticoid use). Model 3 further added health behaviors (smoking, alcohol use). Significant results (p < 0.05) are bolded.*

Abbreviations: AUCg (area under the curve, ground); AUCi (area under the curve, increase)

© 2026 Ng TS et al. *JAMA Network Open*.

eTable 7 — Sensitivity Analyses: Associations Between Salivary Cortisol Quintiles and Global Cognition After Excluding Baseline Participants with the Lowest 10th percentile Global Cognition

eTable 7a. Cross-sectional Associations

CV

Variable	β (95% CI) Model 1	p	β (95% CI) Model 2	p	β (95% CI) Model 3	p
Q2	0.01 (−0.03, 0.05)	0.70	0.02 (−0.03, 0.06)	0.48	0.02 (−0.03, 0.06)	0.45
Q3	**0.05 (0.01, 0.09)**	**0.01**	**0.06 (0.02, 0.10)**	**0.01**	**0.06 (0.02, 0.10)**	**0.01**
Q4	**0.05 (0.01, 0.09)**	**0.02**	**0.05 (0.01, 0.09)**	**0.02**	**0.05 (0.01, 0.09)**	**0.03**
Q5	**0.05 (0.01, 0.09)**	**0.01**	**0.06 (0.02, 0.10)**	**0.01**	**0.06 (0.02, 0.10)**	**0.01**

Mean

Variable	β (95% CI) Model 1	p	β (95% CI) Model 2	p	β (95% CI) Model 3	p
Q2	0.03 (−0.01, 0.07)	0.08	0.04 (−0.00, 0.08)	0.07	0.03 (−0.01, 0.07)	0.09
Q3	0.03 (−0.01, 0.07)	0.16	0.03 (−0.01, 0.07)	0.16	0.03 (−0.02, 0.07)	0.22
Q4	0.02 (−0.02, 0.06)	0.34	0.01 (−0.03, 0.05)	0.51	0.01 (−0.03, 0.05)	0.58
Q5	−0.03 (−0.07, 0.01)	0.13	−0.02 (−0.07, 0.02)	0.26	−0.03 (−0.07, 0.02)	0.20

AUCg

Variable	β (95% CI) Model 1	p	β (95% CI) Model 2	p	β (95% CI) Model 3	p
Q2	0.04 (−0.00, 0.08)	0.08	0.03 (−0.01, 0.08)	0.11	0.03 (−0.01, 0.07)	0.13
Q3	0.04 (−0.00, 0.08)	0.09	0.04 (0.00, 0.08)	0.04	0.04 (−0.00, 0.08)	0.06

© 2026 Ng TS et al. *JAMA Network Open*.

Q4	−0.01 (−0.05, 0.03)	0.67	−0.01 (−0.05, 0.03)	0.64	−0.01 (−0.05, 0.03)	0.62
Q5	−0.03 (−0.07, 0.01)	0.12	−0.03 (−0.07, 0.01)	0.19	−0.03 (−0.07, 0.01)	0.16

Slope

Variable	β (95% CI) Model 1	p	β (95% CI) Model 2	p	β (95% CI) Model 3	p
Q2	−0.02 (−0.06, 0.02)	0.44	−0.02 (−0.06, 0.03)	0.43	−0.01 (−0.05, 0.03)	0.54
Q3	−0.01 (−0.05, 0.03)	0.72	−0.0013 (−0.04, 0.04)	0.95	0.00 (−0.04, 0.04)	0.9985
Q4	**−0.04 (−0.08, 0.00)**	**0.0485**	**−0.05 (−0.09, 0.00)**	**0.04**	−0.04 (−0.08, 0.00)	0.06
Q5	**−0.07 (−0.11, −0.03)**	**< 0.001**	**−0.07 (−0.11, −0.02)**	**0.0026**	**−0.06 (−0.10, −0.02)**	**0.0048**

AUCi

Variable	β (95% CI) Model 1	p	β (95% CI) Model 2	p	β (95% CI) Model 3	p
Q2	−0.00 (−0.04, 0.04)	0.87	−0.01 (−0.05, 0.03)	0.62	−0.01 (−0.05, 0.03)	0.64
Q3	0.01 (−0.04, 0.04)	0.77	0.00 (−0.04, 0.04)	0.85	0.01 (−0.03, 0.04)	0.75
Q4	−0.01 (−0.05, 0.03)	0.64	−0.02 (−0.06, 0.02)	0.38	−0.02 (−0.06, 0.02)	0.46
Q5	**−0.09 (−0.13, −0.04)**	**< 0.001**	**−0.08 (−0.13, −0.04)**	**< 0.001**	**−0.08 (−0.12, −0.04)**	**< 0.001**

Notes: Model 1 controlled for demographics (age, sex, education, and race). Model 2 additionally included health factors (chronic conditions, BMI, APOE ε4, antidepressant and glucocorticoid use). Model 3 further added health behaviors (smoking, alcohol use). Significant results ($p < 0.05$) are bolded.

Abbreviations: AUCg (area under the curve, ground); AUCi (area under the curve, increase)

© 2026 Ng TS et al. *JAMA Network Open*.

eTable 7b. Longitudinal Associations

CV

Variable	β (95% CI) Model 1	p	β (95% CI) Model 2	p	β (95% CI) Model 3	p
Q2	0.01 (−0.01, 0.02)	0.47	0.01 (−0.01, 0.02)	0.41	0.01 (−0.01, 0.02)	0.48
Q3	0.01 (−0.01, 0.02)	0.43	0.01 (−0.01, 0.02)	0.35	0.01 (−0.01, 0.02)	0.36
Q4	0.01 (−0.01, 0.02)	0.25	0.01 (−0.01, 0.02)	0.29	0.01 (−0.01, 0.02)	0.30
Q5	0.01 (−0.01, 0.02)	0.32	0.0045 (−0.01, 0.02)	0.51	0.0047 (−0.01, 0.02)	0.49

Mean

Variable	β (95% CI) Model 1	p	β (95% CI) Model 2	p	β (95% CI) Model 3	p
Q2	**−0.01 (−0.02, 0.00)**	**0.04**	−0.01 (−0.02, 0.00)	0.07	−0.01 (−0.02, 0.00)	0.07
Q3	**−0.01 (−0.02, 0.00)**	**0.05**	−0.01 (−0.02, 0.00)	0.09	−0.01 (−0.02, 0.00)	0.12
Q4	**−0.01 (−0.02, 0.00)**	**0.0479**	−0.01 (−0.02, 0.00)	0.10	−0.01 (−0.02, 0.00)	0.12
Q5	**−0.01 (−0.03, 0.00)**	**0.03**	**−0.01 (−0.03, 0.00)**	**0.03**	**−0.01 (−0.03, 0.00)**	**0.03**

AUCg

Variable	β (95% CI) Model 1	p	β (95% CI) Model 2	p	β (95% CI) Model 3	p
Q2	−0.01 (−0.02, 0.01)	0.39	−0.0042 (−0.02, 0.01)	0.47	−0.0042 (−0.02, 0.01)	0.47
Q3	−0.01 (−0.02, 0.00)	0.24	−0.01 (−0.02, 0.00)	0.21	−0.01 (−0.02, 0.00)	0.27

© 2026 Ng TS et al. *JAMA Network Open*.

Q4	−0.0032 (−0.01, 0.01)	0.61	−0.0010 (−0.01, 0.01)	0.87	−0.0010 (−0.01, 0.01)	0.88
Q5	−0.01 (−0.02, 0.00)	0.09	−0.01 (−0.02, 0.00)	0.08	−0.01 (−0.02, 0.00)	0.10

Slope

Variable	β (95% CI) Model 1	p	β (95% CI) Model 2	p	β (95% CI) Model 3	p
Q2	−0.01 (−0.02, 0.01)	0.39	−0.0022 (−0.01, 0.01)	0.72	−0.0032 (−0.01, 0.01)	0.59
Q3	−0.01 (−0.02, 0.00)	0.20	−0.01 (−0.02, 0.00)	0.22	−0.01 (−0.02, 0.00)	0.22
Q4	−0.01 (−0.02, 0.01)	0.35	−0.0038 (−0.01, 0.01)	0.56	−0.0038 (−0.01, 0.01)	0.55
Q5	−0.01 (−0.02, 0.00)	0.14	−0.01 (−0.02, 0.00)	0.28	−0.01 (−0.02, 0.00)	0.27

AUCi

Variable	β (95% CI) Model 1	p	β (95% CI) Model 2	p	β (95% CI) Model 3	p
Q2	0.01 (−0.01, 0.02)	0.50	0.00 (−0.01, 0.02)	0.55	0.00 (−0.01, 0.02)	0.60
Q3	−0.00 (−0.01, 0.01)	0.54	−0.00 (−0.01, 0.01)	0.65	−0.00 (−0.01, 0.01)	0.56
Q4	0.01 (−0.01, 0.02)	0.32	0.01 (−0.01, 0.02)	0.26	0.01 (−0.01, 0.02)	0.30
Q5	0.01 (−0.01, 0.02)	0.30	0.01 (−0.01, 0.02)	0.31	0.01 (−0.01, 0.02)	0.34

Notes: Model 1 controlled for demographics (age, sex, education, and race). Model 2 additionally included health factors (chronic conditions, BMI, APOE ε4, antidepressant and glucocorticoid use). Model 3 further added health behaviors (smoking, alcohol use). Significant results ($p < 0.05$) are bolded.

Abbreviations: AUCg (area under the curve, ground); AUCi (area under the curve, increase)

© 2026 Ng TS et al. *JAMA Network Open*.
