# Supplementary material for: Salivary Cortisol and Cognitive Decline and Alzheimer Disease in Older Adults
Source: JAMA Netw Open. 2026 Jul 15;9(7):e2622955. doi: 10.1001/jamanetworkopen.2026.22955 (PMC13373666; doi:10.1001/jamanetworkopen.2026.22955)
Supplement: Supplement 2. — Data Sharing Statement [file jamanetwopen-e2622955-s002.pdf]

Data Sharing Statement

Ng. Salivary Cortisol and Cognitive Decline and Alzheimer Disease in Older Adults. *JAMA Netw Open*. Published July 15, 2026. doi:10.1001/jamanetworkopen.2026.22955

Data
Data available: Yes
Data types: Deidentified participant data
How to access data: Rush website (details see manuscript)
When available: With publication

Supporting Documents
Document types: None

Additional Information
Who can access the data: NA
Types of analyses: NA
Mechanisms of data availability: NA
